# Block-and-Lock Approaches for HIV Cure: Mechanistic Insights, Challenges, and Emerging Role of CPSF6

**DOI:** 10.3390/ijms27083496

**Published:** 2026-04-14

**Authors:** Manlio Tolomeo, Antonio Cascio

**Affiliations:** 1 Department of Health Promotion Sciences, Maternal and Infant Care, Internal Medicine and Medical Specialties, University of Palermo, 90127 Palermo, Italy; antonio.cascio03@unipa.it; 2 Department of Infectious Diseases, Azienda Ospedaliera Universitaria Policlinico (A.O.U.P.) Palermo, 90127 Palermo, Italy

**Keywords:** HIV-1, block-and-lock, CPSF6, LEDGF/p75, proviral integration, LADs, functional cure

## Abstract

The block-and-lock strategy aims to achieve a functional cure for human immunodeficiency virus type 1 (HIV-1) infection by enforcing durable, drug-independent silencing of proviral transcription. Several latency-promoting agents have been described that effectively limit viral reactivation in vitro or in animal models. However, most approaches induce only partial or reversible transcriptional repression and have not yet been translated into safe and effective clinical interventions. This review summarizes the molecular mechanisms underlying block-and-lock strategies and critically evaluates the limitations of current candidate compounds. We highlight recent advances in understanding HIV-1 integration site selection, focusing on the roles of lens epithelium-derived growth factor p75 (LEDGF/p75) and cleavage and polyadenylation specificity factor subunit 6 (CPSF6) in directing proviral integration toward gene-dense, transcriptionally active chromatin. Pharmacological disruption of the LEDGF/p75–integrase interaction by LEDGF/p75 inhibitors (LEDGINs) redirects proviral integration toward less transcriptionally active genomic regions that are more resistant to reactivation. Recent tandem knockout experiments, however, demonstrate that CPSF6 plays a dominant role in guiding HIV-1 integration toward gene-dense, transcriptionally active chromatin. LEDGIN treatment has been linked to the preferential targeting of proviruses to heterochromatin-rich regions within the nuclear interior. By contrast, CPSF6 knockout redirects integration toward peripheral heterochromatin, especially lamina-associated domains (LADs), genomic regions typically exhibiting stronger and more stable transcriptional repression than interior heterochromatin. These findings suggest that therapeutic modulation of CPSF6 may exert a more profound and durable effect on proviral silencing within a block-and-lock framework. Nevertheless, complete CPSF6 ablation is associated with severe cellular toxicity. The challenges associated with CPSF6-related adverse effects and potential strategies to overcome these limitations are discussed.

## 1. Introduction

Combination antiretroviral therapy (cART) has profoundly improved the management of human immunodeficiency virus type 1 (HIV-1) infection, transforming it from a fatal disease into a chronic condition. Nevertheless, HIV-1 infection is associated with marked immune dysfunction, including progressive CD4+ T-cell depletion, chronic immune activation, dendritic cell dysregulation, immune cell exhaustion, and alterations in B-cell compartments, as well as perturbations of key signaling pathways such as the signal transducer and activator of transcription (STAT) system [1,2,3]. Some of these immune dysfunctions can persist despite cART or complete restoration of CD4+ T cells. Although cART effectively suppresses viral replication to undetectable levels, it does not eliminate the pool of integrated HIV-1 DNA (provirus) that persists in long-lived immune cells, which remains a major barrier to achieving a definitive cure [4,5].

In the context of HIV-1 infection, a curative intervention can be defined as a strategy capable of achieving durable control or eradication of the virus following a single or limited course of treatment, without the need for ongoing therapy and in the absence of viral rebound after treatment interruption. Importantly, interventions that require continuous or repeated administration to maintain viral suppression are more appropriately classified as therapeutic rather than curative.

An initial attempt to achieve a cure for HIV-1 infection was based on the “shock-and-kill” approach, which aims to reactivate the latent virus and subsequently eliminate infected cells [6,7,8,9,10]. However, limited clinical success has shifted attention toward alternative therapeutic paradigms. In this context, “block-and-lock” strategies seek to achieve a functional cure by enforcing durable viral latency through stable epigenetic silencing of the integrated provirus, thereby preventing viral reactivation even after cART interruption [11,12,13,14,15]. Nevertheless, whether currently available block-and-lock agents can meet the criteria of a true curative intervention remains an open question.

The “block-and-lock” approach relies on agents that inhibit viral transcription (“block”) and promote durable latency (“lock”) through distinct but complementary mechanisms, including: (i) direct inhibition of viral transcription; (ii) post-transcriptional inhibition of HIV-1 gene expression; (iii) inhibition of host transcription factors that activate the HIV-1 LTR; (iv) blockade of cellular kinases and signaling pathways required for HIV-1 transcription; and (v) epigenetic silencing of the provirus and promotion of its integration into repressive chromatin domains (Table 1).

HIV-1 transcription inhibitors, such as didehydro-cortistatin A (dCA), and host transcription factor modulators, such as bromodomain-containing protein 4 (BRD4) modulators, have been extensively studied [16,17,18,19]. Transcriptional kinase inhibitors (CDK8/19 inhibitors) have shown promise in promoting HIV-1 latency and suppressing reactivation in cell models [20,21]. Lens epithelium-derived growth factor (LEDGF/p75) inhibitors (LEDGINs) retarget HIV-1 integration toward less transcriptionally active chromatin, thereby expanding the reservoir into a more deeply latent state. Multiple studies have demonstrated their ability to reduce reactivation potential and promote durable silencing [22,23,24,25].

Although each of these molecular approaches has generated compelling preclinical evidence, none has yet met the safety, tolerability, or formulation criteria required to advance into human clinical trials. Challenges include compound toxicity, off-target chromatin effects, and the absence of delivery systems suitable for long-term administration. As a result, the clinical translation of “block-and-lock” agents remains limited, and there is growing interest in identifying alternative mechanisms that can achieve durable proviral silencing using established therapeutic platforms. Moreover, many of these compounds require sustained exposure to maintain their effects, raising important questions about whether they can achieve irreversible or long-lasting proviral silencing, consistent with a curative outcome. An additional limitation is that several of these approaches rely on modulation of host factors. While transient targeting of host pathways may be acceptable in short-term interventions, strategies requiring prolonged or irreversible alteration of host factor activity raise important safety concerns and are unlikely to be compatible with curative applications. Notably, for compounds with demonstrated block-and-lock activity in vivo in animal models, the time to viral rebound after treatment cessation remains relatively short, ranging from approximately 2 to 4 weeks.

Although these compounds are widely regarded in the literature as block-and-lock agents, their inability to establish a definitive, irreversible “lock” state currently precludes their classification as curative interventions. In most cases, their effects appear to depend on continued drug exposure, and viral reactivation occurs upon treatment discontinuation. However, it should also be acknowledged that in vivo and clinical data remain limited, and the long-term efficacy of these compounds in establishing durable proviral silencing remains unclear.

In contrast, strategies that can redirect proviral integration toward transcriptionally silent chromatin regions may offer a mechanistically distinct and potentially more durable route to a functional cure. Over prolonged treatment periods, such approaches could progressively enrich the pool of infected cells with proviruses embedded in deeply repressive chromatin environments, resulting in a cellular reservoir largely composed of transcriptionally inactive proviruses. In this scenario, following treatment interruption, the lack of transcriptionally competent proviruses could significantly reduce—or potentially eliminate—the likelihood of viral rebound, thereby approximating a functional cure.

Such an effect may be attainable through the use of LEDGINs. However, recent evidence indicates that modulation of CPSF6 expression—either through genetic knockout or knockdown approaches, or through compounds that disrupt CPSF6–capsid interactions—can redirect proviral integration toward highly transcriptionally repressive chromatin regions, specifically LADs, which are typically associated with stable and long-term gene silencing [26,27,28,29].

Notably, two LEDGINs, STP0404 (pirmitegravir) and BDM-2, have advanced to Phase I and II clinical trials, whereas the capsid inhibitor lenacapavir is already approved for the treatment of HIV-1 infection [30,31,32,33,34,35]. This contrasts with most other agents investigated within the block-and-lock framework, for which the absence of comprehensive pharmacokinetic, metabolic, and long-term toxicity data remains a major obstacle to clinical translation. Notably, while these compounds show promise as long-acting or mechanistically innovative antiretroviral agents, their ability to induce a self-sustaining, treatment-independent state of viral silencing consistent with a true cure has yet to be demonstrated. A further limitation of integration-targeting approaches is the requirement for drug availability at the time of viral entry and integration, a process that is inherently stochastic and difficult to predict in vivo, particularly given the limited and sporadic reactivation of the viral reservoir.

In this review, we summarize the current progress achieved with the most relevant compounds evaluated for block-and-lock strategies, with particular emphasis on integration-targeting approaches. The final section provides an in-depth discussion of the therapeutic potential and challenges associated with pharmacological modulation of proviral integration via CPSF6 targeting, with particular consideration of its implications for the development of truly curative interventions.

**Table 1:** Classification of “block-and-lock” therapeutic strategies for HIV-1 infection according to their primary mechanism of action.

Group	Block-and-Lock Agent’s Name or Strategy Name	Mechanism/Target	References
Direct suppressors of HIV-1 transcriptional activity	Tat inhibitors, Triptolide, camptothecin analogs, CRISPR–Cas systems.	Direct inhibition of viral transcription, splicing, or RNA stability.	[16,36,37,38,39,40,41,42,43,44,45,46,47,48,49,50,51,52,53,54,55,56,57,58,59,60,61,62,63,64]
Post-transcriptional agents	Splicing inhibitors, RNA interference technologies.	Sequence-specific silencing of HIV-1 gene expression through RNA degradation or targeted transcriptional repression, promoting durable viral latency.	[65,66,67,68,69,70,71,72,73,74,75,76]
Cellular transcription factor modulators.	BRD4 modulators, NF-κB inhibitors.	Inhibition of host transcription factors that activate HIV-1 LTR.	[77,78,79,80,81,82,83,84,85,86,87,88,89,90,91,92,93,94,95,96,97,98,99,100,101,102]
Cellular Kinase inhibitors.	PI3K–AKT–mTOR pathway modulators, Aurora kinase and PAK1/2 inhibitors, PKC inhibitors, CDK inhibitors, SR kinase inhibitors.	Block of cellular kinases/signaling required for HIV-1 transcription.	[103,104,105,106,107,108,109,110,111,112]
Epigenetic, chromatin, and integration site modulators.	H3K27 demethylase inhibitors, Histone acetyltransferase (HAT) inhibitors, FACT targeting compounds, HIV Antisense RNA (AST/ASP RNA), LEDGF/p75–integrase inhibitors (LEDGINs)	Induction of repressive chromatin and epigenetic silencing of provirus. Integration in transcriptionally silent chromatin	[113,114,115,116,117,118,119,120,121,122,123,124,125,126,127,128,129,130,131,132,133,134,135,136,137,138,139,140,141,142,143,144,145,146,147,148,149,150,151,152,153,154,155,156,157]

## 2. Direct Suppression of HIV-1 Transcriptional Activity

### 2.1. Tat-Mediated Control of HIV-1 Transcription as a Therapeutic Target for Block-and-Lock Strategies

Trans-activator of transcription (Tat) protein inhibitors represent a promising approach within the HIV-1 “block-and-lock” therapeutic paradigm, which aims to achieve durable transcriptional silencing and prevent viral reactivation from latent reservoirs. The Tat protein in HIV-1 is essential for efficient viral gene expression. Its primary function is to strongly enhance transcriptional elongation from the HIV-1 LTR promoter [158,159].

The HIV-1 genome is transcribed by RNA polymerase II (RNAPII) using the 5′ LTR as a promoter. Several cellular transcription factors, including NF-κB, NFATc, Sp1, and TATA-binding protein (TBP), bind to the 5′ LTR and recruit the preinitiation complex (PIC), positioning RNAPII at the transcription start site.

**Figure 1:**
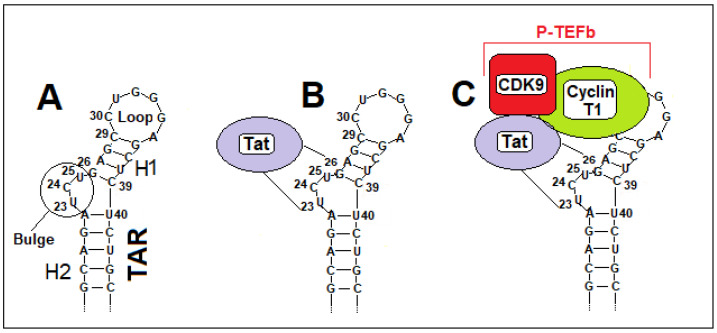
Representative structures of TAR RNA alone (**A**) and in complex with Tat (**B**), and with the Tat–P-TEFb complex (**C**). HIV-1 TAR RNA is organized into two helices (H1 and H2) connected by a characteristic three-nucleotide bulge (U23, C24, and U25) and capped by an apical loop of approximately six nucleotides (typically residues 30–35) (**A**). The Tat protein primarily associates with the TAR bulge via its arginine-rich motif (**B**), whereas the cyclin T1 subunit of P-TEFb forms specific, stabilizing interactions with the TAR loop (**C**). The CDK9 protein does not bind directly to TAR but forms a complex with cyclin T1 and Tat (**C**), serving as the catalytic subunit of the P-TEFb complex and providing the kinase activity required for transcriptional elongation.

RNAPII synthesizes a ~59-nucleotide non-coding RNA stem-loop structure, called the transactivation response element (TAR), located at the 5′ end of the nascent viral transcript. TAR provides the structural platform for Tat and elongation factor recruitment [160]. The hairpin motif of the HIV-1 TAR RNA is organized into two helices interconnected by a characteristic three-nucleotide bulge and terminated by an apical loop of approximately six nucleotides (Figure 1A). Tat primarily associates with the TAR bulge through its arginine-rich motif (ARM) (Figure 1B). As discussed in more detail below, to perform its functions, Tat must interact with the positive transcription elongation factor b (P-TEFb) complex, composed of cyclin-dependent kinase 9 (CDK9) and cyclin T1, which is essential for the initiation of transcriptional elongation of the viral genome. The cyclin T1 subunit of P-TEFb forms specific and stabilizing interactions with the TAR loop (Figure 1C).

**Figure 2:**
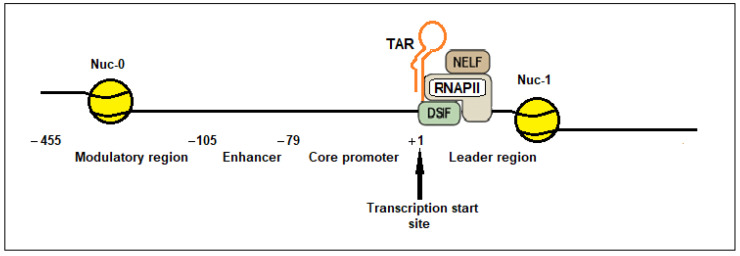
Representative structure of the HIV-1 5′ LTR and organization of nucleosomes and TAR–RNAPII–NELF–DSIF positioning. The 5′ LTR of HIV-1 is organized into four functional domains: the modulatory region, enhancer, core promoter, and leader region. The modulatory region contains binding sites for transcription factors that modulate basal and inducible transcription. The enhancer region typically includes multiple NF-κB binding sites, which are essential for activation in response to cellular signals. The core promoter contains the TATA box and Sp1 binding sites, required for transcription initiation. The leader region, also known as the 5′ untranslated region (5′-UTR), contains additional regulatory elements and is involved in post-transcriptional regulation and RNA processing. The transcription start site is located at the +1 position of the LTR. Nuc-0 and Nuc-1 nucleosomes regulate latency and transcriptional activity of the HIV-1 provirus by controlling chromatin accessibility and the recruitment of transcriptional machinery at the HIV-1 LTR promoter.

TAR is located at the +1 position of the 5′ LTR (Figure 2). The LTR is embedded into two nucleosomes, Nuc-0 and Nuc-1 (Figure 2). The Nuc-0 nucleosome is positioned upstream of the transcription start site, in the modulatory region of the HIV-1 LTR, and specifically spans the modulatory region defined by nucleotides −455 to −105. This region is critical for the epigenetic regulation of HIV-1 latency and transcriptional silencing, as it is subject to histone modifications and chromatin remodeling that influence viral gene expression. Nuc-0, as Nuc-1, consists of an octamer of histone proteins (two copies each of H2A, H2B, H3, and H4). The Nuc-1 nucleosome is located in the leader region of the LTR between +1 and +179 nucleotides, immediately downstream of the transcription start site. It is a key regulatory element for transcriptional activation and latency, as its remodeling is required for efficient transcription initiation and elongation (Figure 2).

Following TAR synthesis, RNAPII pauses near the Nuc-1 nucleosome. This pause is mediated by two promoter-proximal pausing factors: the negative elongation factor (NELF), a multisubunit protein complex found in metazoans that regulates gene transcription, and the DRB sensitivity–inducing factor (DSIF), a conserved transcription elongation factor essential for recruiting NELF to the transcription complex (Figure 2) [161,162].

**Figure 3:**
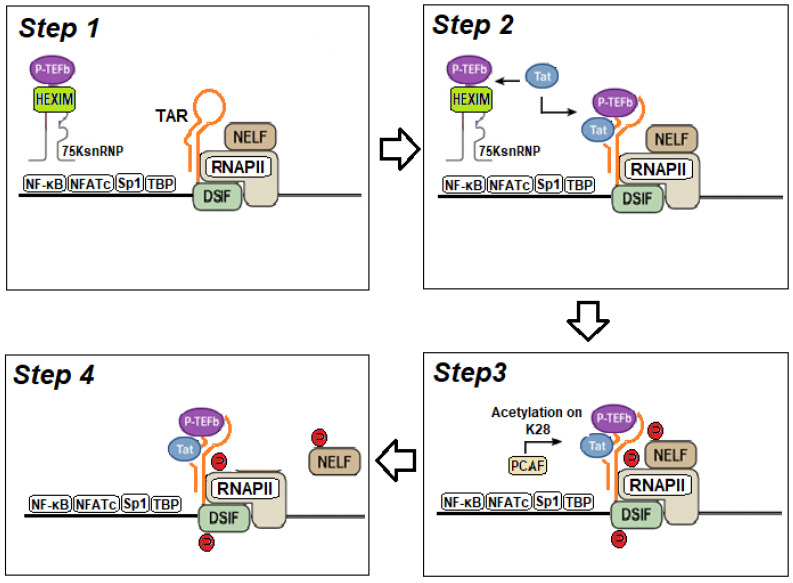
Tat-mediated activation of HIV-1 transcription. Step 1: In HIV-1-infected cells and in the absence of Tat, several cellular transcription factors (TFs)—including NF-κB, NFATc, Sp1, and TBP—bind to the 5′ LTR and promote RNA synthesis. However, RNAPII soon stalls after generating short transcripts due to the recruitment of NELF and DSIF. Step 2: Upon Tat accumulation in the nucleus, this viral protein binds to cyclin T1, a subunit of P-TEFb (a heterodimer composed of CDK9 and cyclin T1), thereby competing with and displacing HEXIM1. This interaction releases the active Tat–P-TEFb complex, which is specifically recruited to the trans-activation response (TAR) RNA element at the 5′ end of nascent HIV-1 transcripts. Step 3: Acetylation of Tat at lysine 28 (K28) by the p300/CBP-associated factor (PCAF) enhances its capacity to recruit P-TEFb. The kinase subunit of P-TEFb, CDK9, phosphorylates both the CTD of RNAPII and the DSIF and NELF complexes. Step 4: These phosphorylation events convert DSIF into a positive elongation factor and promote the dissociation of NELF, allowing RNAPII to transition from a paused to a productive elongation state.

Productive elongation of the viral genome requires the recruitment of P-TEFb [163]. In the absence of Tat, HIV-1 transcriptional elongation is inhibited by the 7SK small nuclear ribonucleoprotein (snRNP)/HEXIM1 complex, which sequesters and inactivates P-TEFb (Figure 3, step 1). This complex is a nuclear ribonucleoprotein assembly that regulates RNA polymerase II–dependent transcriptional elongation and is composed of the noncoding 7SK snRNA and hexamethylene bis-acetamide inducible protein 1 (HEXIM1), which binds both 7SK snRNA and P-TEFb. HEXIM1 is essential for the inhibitory function of the 7SK snRNP, as it directly interacts with the cyclin T1 subunit and the catalytic domain of CDK9, thereby suppressing CDK9 kinase activity. As a result, the 7SK snRNP/HEXIM1 complex acts as a global regulator of P-TEFb activity, modulating transcriptional elongation of both cellular and viral genes.

During HIV-1 infection, Tat competes with the 7SK snRNP/HEXIM1 complex for binding to cyclin T1 of P-TEFb, displacing the inhibitory complex and releasing transcriptionally active P-TEFb [36,164,165,166]. The resulting Tat–P-TEFb complex is subsequently recruited to the TAR RNA element, forming the Tat–P-TEFb–TAR assembly (Figure 3, step 2). The Tat protein primarily associates with the TAR bulge via its ARM (Figure 1B). However, this interaction appears to contribute less to the overall binding affinity than the contacts established between cyclin T1 and the TAR loop. In particular, the Tat–TAR recognition motif (TRM) within cyclin T1 forms specific, stabilizing interactions with the TAR loop (Figure 1C). Tat, in turn, acts as a molecular scaffold that supports and reinforces these contacts, not only through its zinc-coordinating loop but also via electrostatic interactions mediated by the ARM. CDK9 of P-TEFb provides the kinase activity required for phosphorylation of the C-terminal domain (CTD) of RNAPII, DSIF, and NELF. In addition, acetylation of Tat at lysine 28 (K28) by the p300/CBP-associated factor (PCAF) enhances its capacity to recruit P-TEFb. (Figure 3, step 3).

The phosphorylation events mediated by CDK9 convert DSIF into a positive elongation factor and promote the dissociation of NELF, thereby allowing RNAPII to transition from a paused to a productive elongation state (Figure 3, step 4). The newly synthesized Tat protein further amplifies this process by enhancing the recruitment of additional P-TEFb molecules and the Super Elongation Complex (SEC)—a multiprotein assembly composed of P-TEFb, the elongation factors ELL1/ELL2, AF4 family scaffold proteins (AFF1 or AFF4), and either ENL or AF9—to the TAR element, thereby ensuring robust viral RNA synthesis and protein production [160,161,162,163,164,165,166].

Given Tat’s key role in promoting HIV-1 transcription, several classes of Tat inhibitors are currently under active investigation as components of “block-and-lock” strategies aimed at achieving a functional cure for HIV-1 infection.

### 2.2. Didehydro-Cortistatin A: Mechanism and Preclinical Efficacy

**Figure 4:**
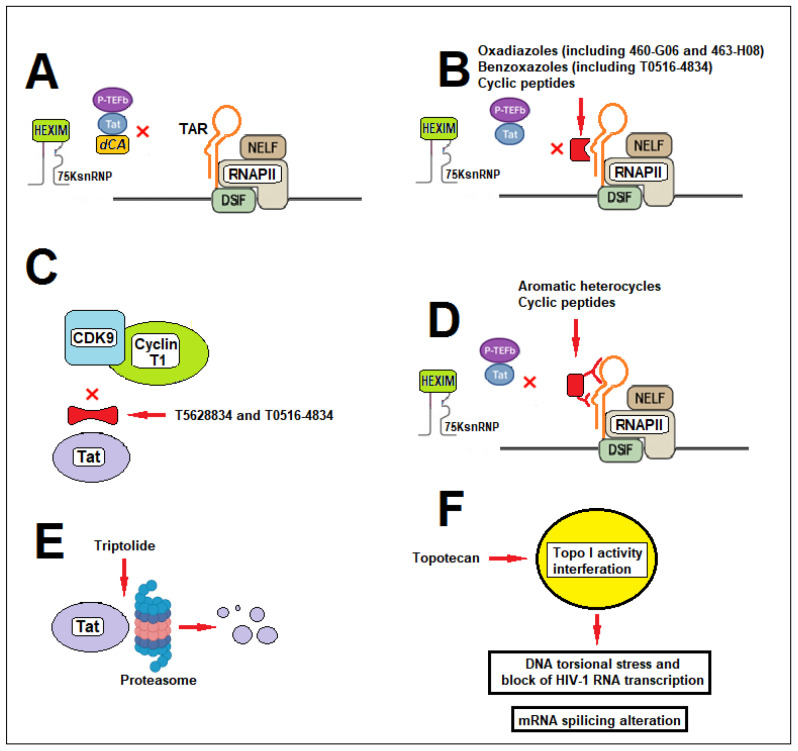
Direct Suppressors of HIV-1 Transcriptional Activity: Molecular Targets and Mechanisms of Action. (**A**) dCA binds directly to the unstructured basic region of Tat, preventing the Tat–P-TEFb complex from recognizing and binding the TAR element. (**B**) Oxadiazole and benzoxazole derivatives inhibit Tat-dependent transcription by binding the TAR bulge, blocking formation of the Tat–TAR complex. (**C**) Compounds T5628834 and T0516-4834 disrupt the Tat–CDK9/cyclin T1 interaction. (**D**) Small molecules and cyclic peptides interact with both the bulge and loop regions of TAR, which is important because the loop is the cyclin T1 binding site within P-TEFb. (**E**) Triptolide promotes proteasome-mediated degradation of Tat, inhibiting viral replication. (**F**) Topotecan suppresses HIV-1 replication by interfering with host Top1 activity, which is required for transcription elongation and also alters mRNA splicing.

Among Tat-targeting compounds, didehydro-cortistatin A (dCA) is the most extensively characterized compound and remains the leading candidate in this class. dCA binds directly to the unstructured basic region of Tat and inhibits the ability of the Tat–P-TEFb complex to recognize and bind the TAR element of viral RNA (Figure 4A). By doing so, it blocks viral transcription, disrupts the transcriptional feedback loop, and promotes epigenetic silencing of the HIV-1 LTR promoter [16,36,37,38,39,40].

In vitro studies have demonstrated that dCA potently suppresses HIV-1 reactivation from latency by inhibiting Tat-mediated transcription. In models of latently infected cells, prolonged treatment with dCA maintained the proviral promoter in a transcriptionally silenced state, and removal of the compound did not result in detectable viral rebound over the observation period, indicating durable repression of viral gene expression [40]. Extending these findings in vivo, Kessing et al. showed that in BLT humanized mice, dCA in combination with cART significantly delayed viral rebound after therapy interruption [16]. Whereas mice receiving cART alone exhibited detectable viremia as early as 3 days post-cART cessation, with all animals rebounding by day 10, in ten mice treated with dCA plus cART, only one had detectable plasma viremia by day 7, three by day 10, and six by day 19 post-therapy interruption (Table 2).

These results illustrate that while dCA can establish persistent transcriptional silencing in vitro, in vivo latency is more complex, and rebound can still occur after cART cessation, albeit significantly delayed. Together, these studies provide strong proof-of-concept that Tat inhibition via dCA contributes to block-and-lock strategies, potentially extending the period of proviral silencing and reducing the frequency of viral reactivation events.

Although dCA is an interesting agent for a block-and-lock approach, resistance may develop [37,42,43]. Resistance is not necessarily due to mutations in the target itself, but rather through heightened basal HIV-1 transcription. In other words, the virus adapts by increasing its basal transcription level, making it less reliant on the Tat protein, which is inhibited by dCA [43]. Of note, dCA-resistant variants can display reduced fitness. Using humanized mice, researchers found that resistant mutants (MUT1/MUT2) exhibited delayed establishment of infection, lower plasma viral loads, and lower proviral DNA levels than wild-type (WT) HIV-1 [42].

### 2.3. dCA Pharmacokinetics, Bioavailability, and Safety

Pharmacokinetic data on dCA remain limited but provide initial insights into its potential for clinical translation. In animal models, dCA has been administered intraperitoneally or subcutaneously, achieving broad systemic distribution and penetration into HIV-1-relevant tissues, including lymph nodes and spleen [17].

dCA exerts potent antiviral activity at nanomolar concentrations; however, data on half-life, metabolism, and oral absorption are lacking, and no formal pharmacokinetic studies reporting plasma concentration–time profiles have been published. Consequently, oral bioavailability remains uncharacterized, and most studies have relied on injectable formulations.

To date, no major toxicity signals have been reported in animal models, although human safety data are unavailable. dCA has not been evaluated in registered clinical trials, nor has it received regulatory approval. Despite its favorable preclinical efficacy and tissue distribution, progress toward clinical application has been limited by incomplete pharmacokinetic characterization, safety concerns, and formulation challenges.

The lack of optimized, stable, and safe formulations—together with potential immunogenicity and insufficient toxicological data—has impeded transition to human trials. Furthermore, certain analogs have shown immune-related adverse effects or other toxicities, underscoring the need for comprehensive safety evaluations. Overall, the absence of detailed pharmacokinetic, metabolic, and long-term toxicity data remains a major barrier to clinical translation [37,38].

Future research should prioritize the development of optimized formulations with improved bioavailability, alongside thorough toxicity, biodistribution, and metabolism studies—including assessments of alternative delivery routes—before advancing to human trials.

### 2.4. Novel Tat Inhibitors and Future Perspectives

Beyond dCA, several new classes of small molecules have been identified as Tat inhibitors within the “block-and-lock” framework. These compounds interfere with Tat-mediated transcription by disrupting critical interactions between Tat and its cofactors—most notably the Tat–TAR RNA and Tat–cyclin T1 complexes—thereby suppressing viral transcription and altering the epigenetic state of the HIV-1 LTR promoter [44,45,46].

Shin et al. developed a homogeneous, mix-and-read time-resolved fluorescence resonance energy transfer (TR-FRET) assay to identify compounds that disrupt the interaction between the HIV-1 Tat protein and the TAR RNA element. By screening a library of 39,360 small molecules, the authors identified two 1,3,4-oxadiazole derivatives, 460-G06 and 463-H08, as the most potent inhibitors of the Tat–TAR interaction. These compounds inhibit Tat-dependent transcription by directly binding to the TAR RNA, thereby preventing the formation of the Tat–TAR complex, which is essential for viral gene expression [44].

Mechanistically, these inhibitors act by targeting the bulge region of TAR RNA, thereby interfering with Tat recognition (Figure 4B). The bulge region plays a pivotal role in mediating the Tat–TAR interaction and constitutes a preferred binding site for small molecules that block Tat-mediated HIV-1 transcription [47,48,49].

In parallel, other small molecules have been developed to target the Tat–TAR RNA interface via rational design, virtual screening, and structure-based docking. Among the most promising candidates, the benzoxazole compound T0516-4834 has been shown to selectively disrupt the Tat–TAR interaction, thereby inhibiting Tat-induced transcription and viral RNA and p24 protein production in HIV-1–infected T cells and PBMCs, without exerting significant effects on cellular transcription [45].

In addition, T0516-4834 disrupted the Tat–CDK9/cyclin T1 interaction. Other molecules, such as compound T5628834, showed a stronger effect in disrupting this interaction (Figure 4C) [45]. Likewise, cyclic peptides and foldamers such as TB-CP-6.9a and ADH-19 exhibit strong affinity for the TAR bulge, thereby reducing viral infectivity in cultured cells without inducing significant cytotoxicity [50,51]. The cyclic peptides described by Chavali et al. selectively engage the major groove of HIV-1 TAR RNA, specifically recognizing guanine bases and the phosphate backbone through their arginine-fork motif. This interaction is primarily localized to the region encompassing the three-nucleotide bulge and the upper helix, the very site where Tat normally binds to mediate transcriptional activation [50].

Recently, Khatkar et al. demonstrated that small molecules targeting the HIV-1 TAR RNA can induce a conformational shift of the TAR loop toward cyclin T1 within the P-TEFb complex, which is essential for efficient HIV-1 transcription [52]. Both the small molecules described by Khatkar et al. and the cyclic peptides reported by Davidson et al. [53] can directly interact with the bulge and loop regions of TAR (Figure 4D). This dual interaction is particularly relevant, as the TAR loop serves as the binding site for cyclin T1 within the P-TEFb complex, a critical component of productive viral transcription. Both studies emphasize that simultaneous targeting of the bulge and loop is a key requirement to achieve complete inhibition of HIV-1 transcription, since interaction with the bulge alone is insufficient to prevent P-TEFb recruitment and Tat function.

Despite these promising results, none of these inhibitors have been evaluated in vivo in animal models, and no clinical data are currently available. Furthermore, no experimental data are currently available regarding viral rebound following removal of the compounds from the culture medium (Table 2). Their assessment, therefore, remains confined to biochemical and cellular models, underscoring the need for further preclinical development and rigorous in vivo validation.

Collectively, these findings highlight the TAR bulge as a highly druggable RNA structural motif and a promising target for the development of novel antiviral agents that can disrupt Tat-dependent HIV-1 transcription. However, for newly identified molecules targeting the Tat-TAR interaction to be truly effective within a “block-and-lock” strategy, they must not only achieve potent inhibition of viral transcription (“block”) but also ensure durable suppression of viral reactivation upon treatment interruption (“lock”). Achieving this latter effect remains particularly challenging for Tat-TAR binding inhibitors, as viral rebound may still occur—albeit with a delay—once drug administration is discontinued, resulting in only a partial, reversible “lock”.

### 2.5. Triptolide-Mediated Tat Degradation as a Block-and-Lock Strategy for HIV-1

Triptolide is a diterpenoid epoxide isolated from *Tripterygium wilfordii* Hook F, a medicinal plant traditionally used for its immunosuppressive and anti-inflammatory properties. In the context of HIV-1 research, triptolide has been shown to potently inhibit viral replication in vitro by promoting proteasome-mediated degradation of the viral transactivator Tat [54] (Figure 4E).

Beyond Tat degradation, triptolide exerts broader transcriptional effects. At the molecular level, it inhibits XPB, a critical helicase subunit of the transcription factor IIH (TFIIH) complex, thereby interfering with global transcriptional processes. In addition, triptolide modulates key cellular signaling pathways involved in HIV-1 transcriptional activation, including NF-κB signaling. Although these pleiotropic activities may further contribute to viral transcriptional repression, they are also associated with significant cytotoxicity. This toxicity represents a major obstacle to therapeutic development and reflects the compound’s narrow therapeutic window [55].

Despite its promising in vitro antiviral activity, there is currently no clinical evidence supporting the efficacy or safety of triptolide as a block-and-lock agent in humans. Moreover, in vivo studies have yielded limited and largely disappointing results. The most relevant data derive from studies using (5R)-5-hydroxytriptolide (LLDT-8), a structural analog with reduced cytotoxicity, in simian immunodeficiency virus (SIV)-infected rhesus macaques, a well-established model of HIV-1 infection.

In SIV-infected macaques receiving cART, LLDT-8 administration was associated with a significant reduction in immune activation markers, including HLA-DR and CD38 co-expression on CD8+ T cells, as well as downregulation of proliferation-related gene pathways in peripheral blood mononuclear cells. However, these immunomodulatory effects did not translate into virological benefit. LLDT-8 failed to reduce viral reservoir size, did not prevent viral rebound after cART discontinuation (Table 2), and did not consistently decrease plasma inflammatory markers or T cell activation levels across studies [56,57].

Overall, while triptolide remains a valuable experimental tool for dissecting mechanisms of HIV-1 transcriptional regulation and latency maintenance, its unfavorable toxicity profile and the absence of convincing in vivo efficacy currently preclude its advancement as a clinical candidate for block-and-lock–based functional cure strategies.

### 2.6. Camptothecin Analogs

Topoisomerase I (Top I) inhibitors, such as the camptothecin analog topotecan, primarily suppress HIV-1 replication by interfering with host Top1 activity, which is essential for relieving torsional stress during transcription elongation. By stabilizing the DNA–Top1 cleavage complex, these compounds impede efficient transcription of the integrated provirus, reducing viral RNA production and downstream protein synthesis. In addition, because HIV-1 RNA splicing is co-transcriptional, the slowed elongation caused by Top1 inhibition can indirectly alter splice-site usage, leading to changes in the ratio of fully spliced, partially spliced, and unspliced viral transcripts. This disruption further limits the production of essential viral proteins and decreases susceptibility to reactivation [58] (Figure 4F).

There are no in vivo studies investigating the effects of camptothecin analogs in the context of HIV-1 infection in the medical literature. All available data on topotecan and other camptothecin analogs in HIV-1 infection are derived from in vitro studies using cell lines and primary cells, where these compounds have demonstrated inhibition of HIV-1 replication, promotion of HIV-1 latency, and interference with viral transcription and RNA splicing.

In vitro studies with topotecan have provided preliminary data on the duration of HIV-1 gene expression suppression after treatment cessation. In an experimental in vitro model of HIV-1 latency using the J-Lat 6.3 reporter cell line, treatment with topotecan for 24 h suppressed HIV-1 expression, with suppression lasting at least 3 days (72 h) after the compound was removed from culture (Table 2). During this follow-up period, cells pretreated with topotecan showed reduced induction of HIV-1 expression compared with controls when stimulated with latency-reversing agents [58]. Due to toxicity, topotecan is unlikely to be used clinically for HIV-1, and there is a call for the development and testing of less toxic analogs, but no animal or human studies have been reported.

### 2.7. CRISPR–Cas Technologies as Block-and-Lock Strategies for HIV-1

CRISPR–Cas systems, most notably CRISPR–Cas9, provide a complementary strategy by enabling sequence-specific editing of HIV-1 proviral DNA integrated into the host genome. By inducing double-strand breaks at predefined loci within the provirus, CRISPR–Cas9 can disrupt essential viral genes or excise large proviral fragments, leading to irreversible inactivation and permanent transcriptional silencing. Xu et al. investigated the use of CRISPR-Cas9 genome editing to permanently inactivate latent HIV-1 proviral DNA, providing experimental support for its potential application within a block-and-lock strategy. Using the J-Lat 10.6 latency model, the authors designed multiple single-guide RNAs (sgRNAs) targeting conserved regions of the HIV-1 genome and tested different sgRNA combinations in association with Cas9. Several dual-sgRNA strategies efficiently induced insertions and deletions within proviral DNA, resulting in functional inactivation of the virus. High editing efficiencies were observed, with selected sgRNA pairs achieving proviral disruption rates exceeding 70%, consistent with a stable silencing of HIV-1 transcriptional competence [59].

There are in vivo studies employing CRISPR–Cas technologies in the context of HIV-1 infection, including models aimed at removing or suppressing integrated proviral DNA, which conceptually aligns with block-and-lock strategies (i.e., durable inactivation of viral genomes). Most of these studies, however, focus on proviral excision or disruption rather than transcriptional silencing alone.

A key study demonstrated that an AAV-delivered multiplex CRISPR-Cas9 system (SaCas9 with multiple guide RNAs) could excise integrated HIV-1 proviral DNA in vivo in HIV-1 transgenic mice (Tg26), EcoHIV-1-infected mice, and HIV-1-infected humanized BLT mice. Following a single intravenous injection of AAV carrying the CRISPR components, proviral excision was detectable in multiple organs, including the spleen, lungs, and brain, and viral RNA expression was significantly reduced both in tissues and systemically. This study provided one of the first demonstrations that CRISPR-mediated proviral genome editing can occur in vivo and reduce viral burden [60].

More recent studies suggest that CRISPR gene editing, combined with cART, in humanized mice can decrease HIV-1 DNA and RNA levels, and, in some animals, a persistent absence of viral signal was observed in multiple tissues over time [61]. In a preclinical non-human primate study, Burdo et al. demonstrated the safety, biodistribution, and in vivo proviral DNA editing using a CRISPR-Cas9 gene editing approach in ART-treated, virally controlled rhesus macaques infected with simian immunodeficiency virus (SIV) [62].

In another study, treatment of HIV-1-infected humanized mice with cART, followed by dual CRISPR-Cas9 targeting of CCR5 and HIV-1 proviral DNA, led to sequential viral suppression, restoration of absolute human CD4+ T-cell numbers, and elimination of replication-competent virus in 58% of infected animals [63].

Preliminary early clinical data from participants receiving the CRISPR-based therapy EBT-101 indicate that, upon discontinuation of cART, most individuals experienced HIV-1 rebound within a few weeks (~2–4 weeks), although one participant maintained viral suppression for approximately 16 weeks [64].

Collectively, these findings highlight the potential of CRISPR-based approaches as a block-and-lock strategy for HIV-1, while underscoring the need for improved editing efficiency and comprehensive targeting of reservoirs to achieve long-term viral remission. Importantly, further clinical studies are required to confirm these preliminary observations.

## 3. Post-Transcriptional and Gene-Silencing Approaches

### 3.1. Splicing Inhibitors

Control of HIV-1 RNA splicing is essential for viral replication, as it enables the regulated production of distinct mRNA species and proteins at specific stages of the viral life cycle. Splicing inhibitors target host cell components of the spliceosome, the molecular machinery responsible for processing RNA transcripts. One key target is splicing factor 3B subunit 1 (SF3B1), a protein that plays a central role in HIV-1 RNA splicing and is required for efficient viral gene expression. Both pharmacological inhibition and genetic depletion of SF3B1 impair Tat-dependent HIV-1 transcription and prevent RNA polymerase II recruitment to the HIV-1 promoter. As a result, HIV-1 reactivation from latency is blocked regardless of the latency-reversing agent used. Importantly, SF3B1 inhibitors—such as sudemycin D6—have shown selective activity against HIV-1-infected cells, supporting their potential as block-and-lock agents [65]. There are no in vivo studies investigating the effects of splicing inhibitors targeting SF3B1 within the block-and-lock approach for HIV-1 infection in the medical literature. All available data on SF3B1 inhibitors are limited to in vitro models, where these compounds have demonstrated modulation of alternative splicing, inhibition of HIV-1 transcription, and reactivation from latency (Table 2). However, it should be considered that SF3B1 inhibitors can cause massive aberrant exon skipping affecting genes involved in DNA repair (BRCA1, BRCA2), nonsense-mediated decay, and the splicing process itself. This broad effect on cellular splicing raises concerns about long-term safety in HIV-infected individuals who would require chronic or repeated dosing. SF3B1 inhibitors like sudemycin D6 face substantial translational barriers, including: (1) dose-limiting toxicities and the need for chronic dosing in a non-cancer population; (2) uncertain CNS penetration to reach sanctuary site reservoirs; (3) lack of validated assays to measure block-and-lock efficacy; and (4) uncertainty about whether pharmacological inhibition produces durable epigenetic silencing. These challenges suggest that significant preclinical and early clinical work remains before SF3B1 inhibitors can advance as HIV cure agents.

### 3.2. RNA Interference Technologies as Block-and-Lock Strategies for HIV-1

RNA interference (RNAi) technologies are actively investigated as block-and-lock approaches to achieve durable suppression of HIV-1 proviral transcription and prevent viral reactivation. RNAi relies on small interfering RNAs (siRNAs) or short hairpin RNAs (shRNAs) to induce sequence-specific degradation of HIV-1 RNA transcripts, thereby reducing viral gene expression and replication. RNAi-based strategies can be engineered to simultaneously target multiple viral transcripts or host dependency factors essential for HIV-1 entry and replication, such as CCR5, and have demonstrated potent antiviral activity in preclinical models. However, their long-term efficacy is challenged by the high genetic variability of HIV-1, which facilitates viral escape, as well as by difficulties in achieving efficient, sustained delivery to reservoir tissues and in maintaining long-term expression of RNAi effectors. Combinatorial RNAi approaches targeting multiple conserved viral regions have been proposed to reduce escape and enhance antiviral durability [66,67,68]. To overcome delivery-related limitations, advanced delivery platforms, including viral vectors and non-viral systems such as lipid- and polymer-based nanoparticles, are under development to improve tissue targeting, cellular uptake, and stability of RNAi molecules, thereby enhancing their translational potential [69].

There are in vivo RNAi studies in HIV models demonstrating transcriptional silencing and durable suppression of HIV-1 gene expression, including promoter-targeted shRNAs that can cause epigenetic repression of the HIV-1 LTR. However, these studies do not conclusively demonstrate robust induction of an HIV-1 latent state with proven rebound resistance; i.e., they do not meet the strict definition of inducing deep latency in an animal model, analogous to block-and-lock cures as currently conceptualized [70,71].

Several experiments have used RNAi (siRNA/shRNA) or RNAi-delivery systems to suppress HIV-1 replication in vivo, usually in humanized mouse models of infection. However, these studies do not convincingly demonstrate a durable “deep latency” block-and-lock effect in vivo. shPromA and related promoter-targeting shRNAs delivered via lentiviral vectors can suppress HIV-1 transcription and replication. This approach has been shown in vivo to knock down HIV-1 expression in animal models (e.g., humanized mice or lentivector-modified human cells engrafted into mice) [70].

A promoter-targeted siRNA, LTR-362, designed to induce transcriptional gene silencing (TGS), was tested in vivo using gp120-aptamer-delivery systems in humanized mice. These conjugates repressed viral RNA levels in serum compared with controls, but the effect did not clearly demonstrate stable, deep latentization via epigenetic silencing of the reservoir and was interpreted mainly as reduced transcription/post-transcriptional silencing at that time point [72]. These studies do show that RNAi constructs can reduce viral transcription or replication in vivo, but they do not show a durable epigenetic lock on the provirus that would constitute deep latentization in a functional cure sense.

Combinatorial strategies integrating CRISPR–Cas and RNAi have been explored to simultaneously target HIV-1 at both the DNA and RNA levels, yielding additive or synergistic antiviral effects. However, overlapping or closely spaced target sites may accelerate the emergence of viral escape mutants through error-prone DNA repair mechanisms and promote cross-resistance, highlighting the need for careful target selection and design [73,74,75,76].

Despite their strong conceptual appeal, key challenges remain for both RNAi- and CRISPR–Cas–based block-and-lock strategies. RNAi-based block-and-lock strategies face formidable translational barriers: (i) delivery systems cannot efficiently reach all anatomical reservoir sites; (ii) safety concerns include off-target effects, saturation of endogenous RNAi machinery, cardiotoxicity, immune activation, and insertional mutagenesis with viral vectors; (iii) HIV-1’s high mutation rate enables rapid escape from single or even combinatorial RNAi targeting; and (iv) distinguishing durable epigenetic silencing from transient knockdown remains methodologically challenging. These barriers explain why RNAi-based HIV therapies remain largely in preclinical development despite decades of research.

To date, neither approach has progressed to the establishment of standardized clinical dosing regimens, as both remain largely confined to preclinical and early translational stages. Ongoing research efforts are focused on improving specificity, delivery technologies, and the long-term durability of HIV-1 transcriptional silencing [13].

## 4. Cellular Transcription Factor Modulators as Block-and-Lock Agents

HIV-1 transcription is tightly controlled by host transcription factors that, together with the viral transactivator Tat, regulate LTR promoter activity. Within this framework, pharmacological modulation of key cellular transcriptional regulators represents a promising block-and-lock strategy to achieve stable, durable suppression of HIV-1 gene expression. Rather than eliminating the integrated provirus, this approach seeks to enforce a deeply silenced state that prevents viral rebound.

Among the critical factors involved, BRD4 and NF-κB play central roles. BRD4, a chromatin-associated member of the BET protein family, supports basal transcription from the integrated provirus. NF-κB functions as a major inducible activator of the HIV-1 promoter. By integrating pro-inflammatory and cellular activation signals, NF-κB can destabilize latency and promote proviral reactivation.

Targeting BRD4 and NF-κB, therefore, allows interference with critical transcriptional nodes governing HIV-1 expression, promoting a deeper and more durable state of viral latency. Fine-tuning host transcriptional regulatory networks may thus represent a cornerstone for the success of block-and-lock strategies.

### 4.1. BDR4 Functions Under Physiologic Conditions and in HIV-1 Infection

BRD4 is a member of the BET (bromodomain and extra-terminal domain) protein family and contains two bromodomains (BD1 and BD2) that recognize acetylated lysine residues on histone tails. Its primary function is to act as an epigenetic and transcriptional regulator by binding acetylated chromatin and interacting with RNAPII, thereby facilitating transcriptional activation. Histone acetylation involves the addition of an acetyl group (CH_3_CO–) to lysine residues within histone tails, a reaction catalyzed by histone acetyltransferases (HATs) and reversed by histone deacetylases (HDACs). This post-translational modification neutralizes the positive charge of lysine residues, thereby weakening histone–DNA interactions and promoting a more open and accessible chromatin conformation, which is generally associated with transcriptional activation. In addition, histone acetylation functions as a molecular signal for the recruitment of regulatory proteins, including the bromodomain-containing protein BRD4, which acts as a scaffold by binding acetylated histones via its bromodomains, thereby promoting chromatin opening and facilitating the recruitment of transcription factors and coactivators to gene promoters [77,78,79,80,81]. However, BRD4 displays functional versatility and exerts context-dependent effects on HIV-1 transcription, largely shaped by its specific interactions with histones and partner proteins.

Under physiological conditions, BRD4 primarily functions as a transcriptional activator by promoting the release of P-TEFb from the inhibitory 7SK snRNP/HEXIM1 complex. This activity depends on a P-TEFb–interacting domain (PID) in the C-terminus of BRD4, which directly binds P-TEFb and facilitates its dissociation from the inhibitory complex. Once released, P-TEFb becomes transcriptionally active and can be recruited to chromatin, thereby supporting productive RNAPII–mediated transcription [82,83,84,85].

**Figure 5:**
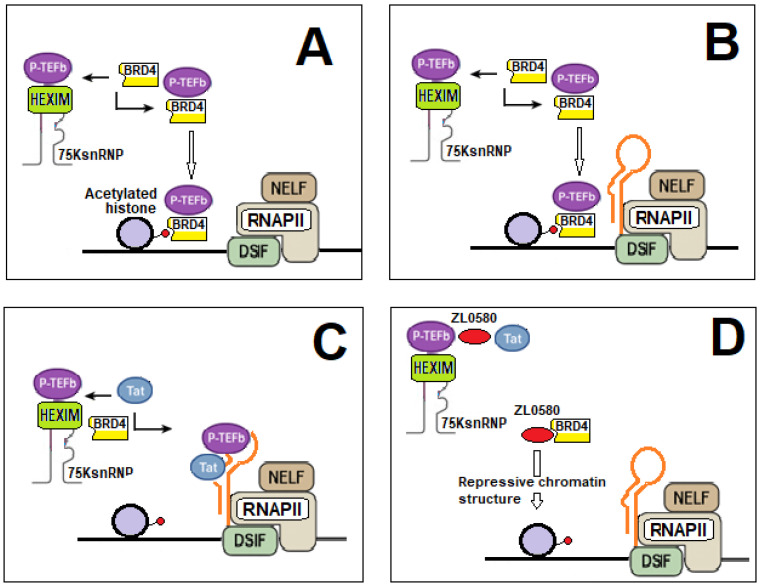
Schematic Representation of BRD Functions Under Physiologic Conditions and in HIV-1 Infection. (**A**) Under physiological conditions, BRD4 acts primarily as a transcriptional activator by promoting the release of P-TEFb from the inhibitory 7SK snRNP/HEXIM1 complex, enabling its recruitment to sites of transcription. BRD4 acts as a scaffold by binding acetylated histones via its bromodomains, promoting chromatin opening and the recruitment of transcription factors and coactivators to gene promoters. In addition, BRD4 facilitates transcriptional elongation primarily by recruiting P-TEFb to chromatin and paused RNAPII complexes. P-TEFb, which contains the kinase CDK9, phosphorylates DSIF, NELF, and the CTD of RNAPII, leading to the dissociation of NELF and conversion of DSIF into a positive elongation factor, thus enabling productive elongation. (**B**) During HIV-1 infection, BRD4 retains the capacity to extract P-TEFb from the inactive 7SK snRNP complex and to recruit it in close proximity to RNAPII. The BRD4-associated P-TEFb complex phosphorylates the CTD of RNAPII, supporting basal transcription from the provirus. Histones H3 and H4 of nucleosomes Nuc-0 and Nuc-1 are the main binding substrates for BRD4, and their acetylation is the key determinant of the interaction with this protein. However, in infected cells, this process is relatively inefficient in the absence of Tat. (**C**) In the presence of Tat, this viral transactivator competes with BRD4 for binding to P-TEFb. Tat displays a higher affinity for the cyclin T1 subunit of P-TEFb than BRD4 and efficiently displaces BRD4 to release P-TEFb from the HEXIM1-containing 7SK snRNP complex, facilitating assembly of the Tat/P-TEFb complex and its recruitment to TAR to drive robust viral transcription. (**D**) ZL0580 binds the BD1 domain of BRD4, inducing a conformational change that promotes repressive chromatin at the HIV-1 LTR, reducing chromatin accessibility, and preventing recruitment of transcriptional activators such as P-TEFb. Additionally, ZL0580 disrupts Tat–CDK9 interactions and inhibits P-TEFb recruitment.

When associated with BRD4, P-TEFb is positioned in close proximity to RNAPII, enabling efficient phosphorylation of the RNAPII CTD, as well as of the negative elongation factor (NELF) and DRB sensitivity–inducing factor (DSIF). This phosphorylation cascade promotes NELF dissociation and converts DSIF into a positive elongation factor, ultimately facilitating productive transcript elongation (Figure 5A).

During HIV-1 infection, BRD4 retains the ability to extract P-TEFb from the inactive 7SK snRNP complex and recruit it near RNAPII. The BRD4-associated P-TEFb complex phosphorylates the RNAPII, thereby supporting basal transcription from the integrated provirus. However, in infected cells, this process remains relatively inefficient in the absence of Tat [86,87,88,89] (Figure 5B).

In the presence of Tat, this viral transactivator directly competes with BRD4 for binding to P-TEFb. Tat exhibits a higher affinity for the cyclin T1 subunit of P-TEFb than BRD4 and efficiently displaces BRD4, promoting the release of P-TEFb from the HEXIM1-containing 7SK snRNP complex. This displacement facilitates assembly of the Tat–P-TEFb complex and its recruitment to the TAR RNA element, driving robust viral transcription [88,89]. The ability of Tat to extract P-TEFb from 7SK snRNP is further enhanced by cofactors such as AFF1, which stabilize Tat–P-TEFb interactions and promote formation of the SEC [88]. Although phosphorylation of specific CDK9 residues, including Ser175, can modulate the relative affinity of BRD4 and Tat for P-TEFb, Tat remains the dominant competitor for P-TEFb in HIV-1–infected cells [90] (Figure 5C).

Following dissociation from the inactive 7SK snRNP/HEXIM1 complex, a transient pool of free P-TEFb is generated. This pool is not immediately bound by Tat or BRD4 and can be rapidly captured by either factor or by additional transcriptional regulators [91]. Tat and BRD4 therefore primarily compete for this free P-TEFb fraction; however, Tat prevails because of its greater binding affinity [86,89].

The proportion of P-TEFb associated with BRD4 during HIV-1 infection has not been precisely quantified. Nonetheless, seminal studies indicate that under physiological conditions, approximately half of the cellular P-TEFb pool is bound to BRD4, whereas the remaining fraction is sequestered within the inactive 7SK/HEXIM1 complex [82,85]. In the presence of Tat, this equilibrium is disrupted: Tat preferentially recruits P-TEFb to sustain viral transcription, thereby reducing its availability for BRD4, although a residual fraction remains BRD4-associated. During HIV-1 latency, BRD4 can retain a substantial proportion of P-TEFb, contributing to the maintenance of transcriptional silencing of the integrated provirus [86,92,93].

### 4.2. Pharmacological Modulation of BRD4

Among the molecules that can interfere with BRD4, two have been extensively studied: JQ1 and ZL0580 [93]. Although the precise mechanisms of action of these compounds remain under investigation and are not yet fully elucidated, studies have shown that they exert opposite effects on HIV-1 infection. The pan-BET inhibitor JQ1 broadly enhances HIV-1 transcription, whereas ZL0580 promotes epigenetic repression of HIV-1 transcription, favoring proviral silencing consistent with a ‘block-and-lock’ approach [19].

JQ1 is a small-molecule inhibitor of the BET protein family, particularly BRD4, developed in 2010. Its discovery marked a turning point in cancer research and epigenetic biology, enabling the pharmacological modulation of BRD4 [94].

The mechanism of action of JQ1 involves competitive binding to the BRD4 bromodomains BD1 and BD2, thereby preventing BRD4 from interacting with acetylated histone tails. This blockade disrupts BRD4 recruitment of the P-TEFb complex, which is required for RNAPII phosphorylation and subsequent transcriptional elongation. As a result, JQ1 represses the transcription of oncogenic genes such as MYC and other targets associated with cellular proliferation and survival [94].

In contrast, during HIV-1 infection, JQ1 activates transcription of the integrated provirus [95,96]. This effect reflects the distinctive regulation of viral transcription: BRD4 competes with Tat for P-TEFb, and JQ1 disrupts this interaction, favoring Tat-mediated transactivation. Under latency conditions, BRD4 can retain a substantial fraction of P-TEFb, contributing to transcriptional silencing of the integrated provirus. Inhibition of BRD4 by JQ1 facilitates P-TEFb recruitment by Tat, and consequently enhances proviral transcriptional elongation [72,73]. Moreover, JQ1 promotes the transient release of P-TEFb from its inactive form (the 7SK snRNP complex), increasing the pool of free P-TEFb available for transcription, including HIV-1 proviral transcription [71]. Therefore, whereas BRD4 is essential for transcriptional elongation of host genes, its inhibition in the context of HIV-1 favors viral transcription due to Tat-mediated activation.

It was recently shown that JQ1 induces dissociation of BRD4 from the repressive chromatin-remodeling proteins (SWI/SNF) at the HIV-1 LTR, thereby reversing BRD4-mediated HIV-1 transcriptional suppression [92]. This is considered independent of Tat-mediated transcription elongation but dependent on the regulatory chromatin structure at the HIV-1 LTR. JQ1 has been explored as a potential agent within a ‘kick-and-kill’ strategy for HIV-1 cure interventions. However, JQ1 is not currently approved for clinical use in humans, and its toxicity profile remains a major obstacle to its translation into routine therapeutic applications, including cancer treatment.

Different from JQ1, ZL0580 selectively represses genes involved in transcriptional activation and chromatin accessibility [97]. This selective targeting of the BRD4 BD1 domain reduces off-target effects and toxicity compared with pan-BET inhibitors that affect all BET family members and both BD1 and BD2 domains.

ZL0580 suppresses HIV-1 transcription through multiple mechanisms. It binds the BD1 domain of BRD4 at Glu-151, inducing a conformational change that promotes repressive chromatin at the HIV-1 LTR, reduces chromatin accessibility, and prevents recruitment of transcriptional activators such as P-TEFb [18,98]. Additionally, ZL0580 disrupts Tat–CDK9 interactions and inhibits P-TEFb recruitment, further blocking transactivation and transcriptional elongation [18,98]. Beyond the viral promoter, it reprograms host chromatin architecture, increasing nucleosome density and reducing accessibility at other genomic loci, contributing to durable silencing. Finally, ZL0580 promotes durable epigenetic silencing by facilitating the recruitment of additional repressive chromatin remodeling complexes, such as the BAF (SWI/SNF) complex, to the HIV-1 LTR, thereby enhancing transcriptional repression [18,98]. ZL0580 is also effective in suppressing HIV-1 in myeloid reservoirs, including microglia and macrophages, which are critical for CNS persistence [99].

The in vivo HIV-1-suppressive activity of ZL0580 was assessed in a humanized mouse model of HIV-1 infection characterized by robust human immune cell reconstitution. Following systemic infection, animals were treated with ZL0580 either as monotherapy or in combination with standard cART, and plasma viral replication was longitudinally monitored. ZL0580 administration led to a rapid and sustained reduction of plasma viremia, comparable to cART, with monotherapy achieving near-undetectable viral loads in vivo. Upon treatment interruption, viral rebound occurred earlier in cART-treated mice, whereas ZL0580-treated animals displayed delayed rebound and lower post-treatment viremia. Specifically, after analytical treatment interruption at week 7, rapid viral rebound occurred in the cART-only group by week 9 (2 weeks post-interruption), whereas the ZL0580 monotherapy group maintained undetectable plasma viral loads at this time point. By week 11 (4 weeks post-interruption), viremia became detectable in both the ZL0580-only and ZL0580 plus cART groups, but at lower levels than in the cART-only group (Table 2). These findings indicate that ZL0580 provided a measurable delay in viral rebound and partial suppression of viremia in vivo, supporting its potential as a block-and-lock agent in HIV-1 cure strategies. All treatment regimens were well tolerated, with no detectable adverse effects [74].

Mechanistically, transcription-factor modulators such as ZL0580 differ from integration-site-targeting strategies because they primarily influence proviral activity without altering its genomic location. ZL0580 can induce durable epigenetic silencing and delay viral rebound, but this effect is likely less permanent than approaches that redirect proviruses into transcriptionally repressive chromatin. In theory, prolonged antiretroviral therapy combined with integration-site modulation could, over time, enrich the reservoir of proviruses stably integrated into repressive chromatin. In this scenario, a potential treatment interruption might not trigger viral rebound, as deeply silenced proviruses would remain transcriptionally inactive.

Importantly, combination strategies using transcription-factor modulators and integration site modulators, such as ZL0580, together with LEDGINs, have demonstrated additive effects in blocking HIV-1 reactivation in both cell lines and primary cells. These findings support the rationale for multi-target “block-and-lock” approaches (the potential mechanisms underlying this additive effect are discussed in Section 6) [18].

No clinical safety data in humans have been published to date for ZL0580, and its translational potential will depend on future phase I studies to confirm the absence of significant off-target or systemic toxicities.

### 4.3. NF-κB Inhibitors as Block-and-Lock Agents in HIV-1 Infection

Inhibitors of nuclear factor kappa B (NF-κB) have been identified as potential agents for block-and-lock strategies in HIV-1 infection. These compounds primarily act by inhibiting the IκB kinase (IKK) signaling pathway, thereby preventing NF-κB activation and subsequent transcription of the HIV-1 provirus. Among the compounds investigated, ACHP is a selective inhibitor of IKKα and IKKβ that has been shown to suppress latent virus reactivation in cellular models by reducing TNF-α–induced HIV-1 gene expression, without significant cytotoxicity at active concentrations [100].

Natural compounds have also been identified, including globospiramine, a spirobisindole alkaloid, which inhibits the NF-κB activation cascade and effectively blocks viral reactivation in vitro [101]. Additional agents, such as antioxidants (N-acetylcysteine, α-lipoic acid) and salicylates (acetylsalicylic acid), have been investigated for their ability to inhibit IκB phosphorylation and degradation, thereby reducing NF-κB activity [102].

Recently, Peters et al. found that treating HIV-1-infected monocyte-derived macrophages with NF-κB inhibitors (caffeic acid or resveratrol) reduced NF-κB activation and modestly accelerated latency establishment. Unexpectedly, however, proviruses in these cells became irreversibly refractory to reactivation by multiple latency reversal agents (LRAs), including LPS and SAHA, even after the inhibitors were removed and NF-κB activity was restored.

Collectively, these NF-κB inhibitors are considered promising candidates for block-and-lock strategies; however, there are no well-established reports yet of NF-κB inhibitors tested in animal models specifically to enforce HIV-1 latency, and their long-term safety and efficacy in the context of HIV-1 infection remain to be fully characterized.

## 5. Kinase Inhibitors in the Block-and-Lock Strategy

Kinase inhibitors are a versatile class of compounds under investigation for their potential to enforce HIV-1 latency in block-and-lock strategies. HIV-1 hijacks multiple cellular kinase pathways to promote transcription, replication, and reactivation from latency, and targeting these kinases can stabilize proviral silencing. Among these, inhibitors of the PI3K–AKT–mTOR pathway, Aurora and p21-activated kinases, protein kinase C (PKC), cyclin-dependent kinases (CDKs), and SR kinases have all been shown to suppress HIV-1 transcription and reduce the likelihood of viral rebound.

The PI3K–AKT–mTOR axis is a central regulator of chromatin structure, transcriptional activation, and cell survival, and HIV-1 exploits this pathway to facilitate reactivation from latency. Small-molecule inhibitors such as ponatinib and ripretinib prevent transcription factor activation at the HIV-1 LTR, limit chromatin accessibility, and reduce RNA polymerase II recruitment, without inducing global T-cell activation or cytotoxicity [103,104]. Aurora kinases and PAK1/2, beyond their canonical roles in mitosis and cytoskeletal dynamics, modulate transcriptional elongation and chromatin remodeling, and their inhibition decreases phosphorylation of transcriptional co-factors, reinforces Polycomb-mediated silencing, and stabilizes repressive histone marks [105,106,107,108,109]. Compounds such as danusertib and PF-3758309 have demonstrated potent suppression of HIV-1 transcription and blockade of latency reversal in vitro, highlighting the utility of targeting kinases involved in transcriptional regulation.

PKC inhibitors, including sotrastaurin and GF109203X, act upstream in signaling cascades required for NF-κB activation, a key driver of HIV-1 transcription, and prevent latency reversal induced by potent PKC agonists [110]. Cyclin-dependent kinases, particularly CDK9, CDK8/19, and CDK7, are central to transcriptional elongation and initiation. While CDK9 inhibitors like LDC000067 suppress proviral expression, latency is not stable after drug withdrawal [111]. By contrast, CDK8/19 inhibitors such as Senexin A and BRD6989 induce deeper and more durable latency in vitro, whereas CDK7 inhibition with YKL-5-124 effectively silences HIV-1 but triggers cell cycle arrest, limiting clinical applicability [111]. Finally, SR kinase inhibitors targeting CLK1/2 regulate alternative splicing and post-initiation RNA processing, stabilizing latency without global T-cell activation and demonstrating nanomolar potency in vitro [112].

Collectively, these observations illustrate that kinase inhibitors can enforce HIV-1 latency through multiple converging mechanisms, including suppression of transcriptional activators, modulation of chromatin accessibility, and interference with post-transcriptional processes. While most evidence remains preclinical, these compounds provide a mechanistic framework for combinatorial block-and-lock strategies. Careful selection of kinase targets, balancing potency and safety, will be crucial for translating these findings into clinically relevant interventions aimed at durable HIV-1 silencing.

## 6. Epigenetic and Chromatin-Based Silencing Approaches Within the Block-and-Lock Strategy

Although the agents described in this chapter act through diverse molecular mechanisms, they all converge on shaping the epigenetic and chromatin landscape of the HIV-1 provirus, thereby modulating transcriptional activity and stabilizing viral latency. These agents include compounds targeting histone-modifying enzymes, chromatin-associated factors that regulate transcriptional elongation, and host determinants of proviral integration site selection. For clarity, they are collectively discussed within the framework of Epigenetic and Chromatin-based silencing approaches. The chapter is organized into three sections: Direct Epigenetic Enzyme Inhibitors (targeting histone-modifying enzymes), Chromatin-Associated Transcriptional Modulators (altering chromatin architecture and elongation dynamics, exemplified by CBL0100 and Q308), and Integration Site Modulators (LEDGINs/ALLINIs, which redirect integration toward chromatin regions less permissive to transcription).

### 6.1. Direct Epigenetic Enzyme Inhibitors

The positioning of Nuc-0 and Nuc-1 nucleosomes at the HIV-1 LTR critically regulates proviral latency by controlling chromatin accessibility and recruitment of transcriptional machinery. Nuc-1, immediately downstream of the transcription start site, acts as a major barrier to transcription initiation and elongation. In latent cells, Nuc-1 is stably positioned and enriched with repressive histone modifications, including H3K9me3 and H3K27me3, which compact chromatin and prevent access by RNA polymerase II, thereby silencing transcription. Nuc-0, upstream of the transcription start site, further contributes to latency by maintaining a repressive chromatin environment, restricting transcription factor binding, and stabilizing silencing. The combined presence of Nuc-0 and Nuc-1 establishes a nucleosomal barrier that enforces proviral latency. Upon cellular activation or exposure to latency-reversing agents, both nucleosomes undergo remodeling, with increased histone acetylation, nucleosome displacement, and enhanced transcriptional activity from the LTR [113,114,115,116,117,118,119,120].

Many direct inhibitors of epigenetic enzymes investigated to date, including H3K27 demethylase inhibitors such as GSK-J4 and histone acetyltransferase (HAT)–targeting agents, have been shown to suppress viral reactivation or induce transient transcriptional repression (Table 2). However, these compounds have not consistently met the stringent requirement for durable, stable silencing that defines a functional “lock” within the block-and-lock strategy. Nevertheless, modulation of histone marks remains a mechanistically compelling and biologically relevant approach within block-and-lock strategies. Given the ongoing development of compounds targeting histone-regulating enzymes, this topic is included in the present review, despite the limited evidence supporting the establishment of a robust, long-lasting functional “lock” with the agents studied so far.

GSK-J4 is a potent dual inhibitor of the H3K27me3/me2 demethylases JMJD3 (KDM6B) and UTX (KDM6A), two epigenetic regulators that remove repressive H3K27 methylation marks from chromatin. Under physiological conditions, JMJD3 and UTX counteract Polycomb-mediated silencing by demethylating H3K27me3 and H3K27me2, thereby promoting transcriptional activation.

In vitro, pharmacological inhibition of these demethylases by GSK-J4 enhanced the repressive chromatin environment, suppressed reactivation of latent HIV-1, and induced DNA methylation at specific CpG sites within the 5′ LTR. However, to date, no histone lysine methyltransferase (HKMT) modulator has achieved durable and permanent HIV-1 silencing within a validated block-and-lock strategy (Table 2) [121].

HATs, including p300/CBP and GCN5, regulate chromatin accessibility by acetylating lysines on histone tails and non-histone proteins, including viral Tat and viral integrase. Acetylation of integrase by p300/GCN5 enhances its DNA-binding, catalytic efficiency, and stability, while HAT-mediated histone acetylation near integration sites creates a permissive chromatin environment, facilitating both integration and transcription. Tat further recruits p300/GCN5 to the LTR, establishing a positive feedback loop that amplifies proviral transcription and viral production.

Several natural compounds, such as curcumin and isogarcinol, inhibit p300/GCN5 activity, though their limited specificity and cytotoxicity restrict their use. Chemical derivatives, including LTK14 (from isogarcinol/garcinol) and coumarin derivatives such as BPRHIV001, exhibit improved selectivity and effectively suppress Tat-mediated transcription in vitro [122,123,124]. These findings underscore HATs as promising targets for HIV-1 block-and-lock strategies, although their ability to prevent latent virus reactivation in vivo remains to be determined, highlighting the need for further mechanistic and translational research.

### 6.2. Chromatin-Associated Transcriptional Modulators

The FACT (Facilitates Chromatin Transcription) complex regulates nucleosome disassembly and reassembly during transcriptional elongation by facilitating RNA polymerase II progression through chromatin. In HIV-1 infection, FACT paradoxically functions as a negative regulator of viral transcription at the LTR, contributing to the establishment and maintenance of proviral latency. This effect is mediated by FACT-dependent preservation of a relatively repressive chromatin environment that interferes with the efficient recruitment and function of cyclin T1 and the viral transactivator Tat, thereby impairing RNA polymerase II processivity and inhibiting HIV-1 transcription [125,126,127].

Small-molecule FACT-targeting compounds, including the curaxin CBL0100 and Q308, have been shown to paradoxically promote HIV-1 latency by blocking viral transcription and preventing reactivation [128,129]. Notably, these effects are not mediated by simple FACT inhibition or depletion, which instead favors viral reactivation, but by functional modulation of FACT and suppression of Tat-dependent transcription.

Q308 was found to inhibit Tat-mediated transcription and selectively downregulate components of the facilitated chromatin transcription (FACT) complex, which is involved in chromatin remodeling and efficient transcriptional elongation of the HIV-1 genome. In multiple in vitro models of HIV-1 latency, Q308 effectively suppressed reactivation of latent virus without significant cytotoxicity. Notably, treatment with Q308 also induced preferential apoptosis in latently infected cells, suggesting a potential to reduce the size of the viral reservoir and thereby further prevent viral rebound. These findings indicate that Q308 operates through a dual mechanism—transcriptional repression and reservoir reduction—supporting its potential as a novel and relatively safe latency-promoting agent (LPA) within block-and-lock strategies aimed at achieving a functional cure for HIV-1 infection [129].

CBL0100 induces chromatin trapping of FACT, reducing its occupancy and that of RNA polymerase II at the HIV-1 LTR and reinforcing viral latency [128,130], whereas genetic FACT depletion leads to chromatin relaxation and spontaneous HIV-1 reactivation [126,131]. Similarly, Q308 suppresses Tat-mediated transcription and downregulates FACT expression, stabilizing latency and increasing the susceptibility of latently infected cells to apoptosis [129].

Overall, these findings indicate that the block-and-lock activity of FACT-targeting agents arises from functional modulation of FACT rather than from its depletion, and from inhibition of Tat-dependent transcription. Although CBL0100 and Q308 represent promising latency-promoting candidates, their translational potential remains unproven, as there are currently no published in vivo studies (in animal models or humans).

### 6.3. Integration Site Modulators

LEDGF/p75–integrase interaction inhibitors, known as LEDGINs, are small molecules that disrupt the interaction between the cellular protein LEDGF/p75 (lens epithelium-derived growth factor) and HIV-1 integrase. They are referred to as allosteric integrase inhibitors (ALLINIs) and can retarget proviral integration toward genomic regions that are less transcriptionally active and less permissive to reactivation [22,24,132,133].

Beyond their well-characterized ability to disrupt the interaction between HIV-1 integrase and LEDGF/p75 during the early phase of infection, LEDGINs exert a major antiviral effect during the late phase of the viral replication cycle. This activity is now recognized as the predominant mechanism underlying their antiviral potency.

During virus production, LEDGINs bind to integrase within assembling virions and induce aberrant multimerization of the enzyme. This altered integrase oligomerization state interferes with its interaction with the viral RNA genome, which is essential for proper ribonucleoprotein (RNP) complex formation. As a consequence, viral particles produced in the presence of LEDGINs exhibit severe structural defects, including mislocalization of the viral ribonucleoprotein complex outside the capsid core. These defects result in the generation of non-infectious virions that cannot support efficient reverse transcription upon infection of target cells.

**Figure 6:**
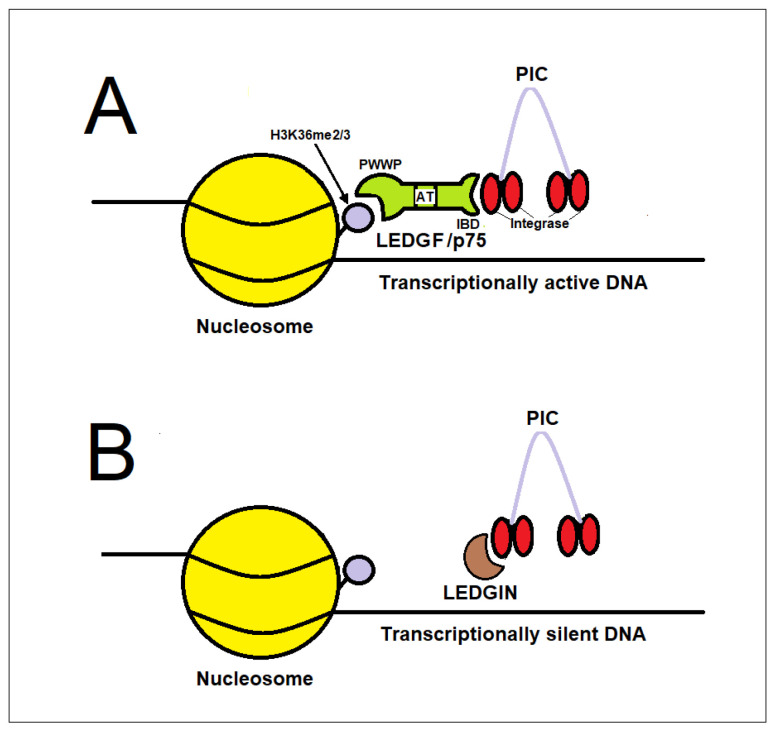
Schematic Representation of LEDGF/p75 Function and Mechanism of Action of LEDGIN in HIV-1 Infection. (**A**) During HIV-1 proviral integration, the PWWP domain of LEDGF/p75 mediates chromatin binding by recognizing H3K36me2/3-enriched nucleosomes, thereby tethering the PIC to transcriptionally active regions of the genome. The IBD of LEDGF/p75 directly interacts with HIV-1 integrase, forming a stable complex that positions the viral PIC at specific chromatin sites and is critical for efficient and site-selective integration. The AT-hook motifs further contribute to DNA binding, strengthening chromatin association and stabilizing the integration complex’s tethering to host DNA. (**B**) LEDGINs act as allosteric integrase inhibitors by binding to the LEDGF/p75 interaction site on integrase, thereby disrupting the integrase–LEDGF/p75 interaction and redirecting proviral integration toward less transcriptionally active genomic regions.

Physiologically, LEDGF/p75 is a nuclear protein that functions as both a transcriptional coactivator and an epigenetic reader. Through its PWWP domain (a protein motif of approximately 100–150 amino acids, characterized by the conserved Pro-Trp-Trp-Pro sequence) LEDGF/p75 recognizes specific histone methylation marks and recruits protein complexes involved in gene regulation, DNA repair, and cellular stress responses promoting cell growth and survival, exerting an anti-apoptotic effect on various cell types, including lens epithelial cells, fibroblasts, and keratinocytes [134]. In HIV-1-infected cells, LEDGF/p75 is a key cellular cofactor of the viral integrase. LEDGF/p75 preferentially directs HIV-1 integration toward chromatin regions enriched in the H3K36me2/3 histone marks, which are associated with active transcription [25,135,136] (Figure 6A). Using multiple complementary approaches, it has been demonstrated that LEDGINs not only reduce overall HIV-1 integration efficiency but also markedly alter the selection of integration sites.

Mechanistically, these compounds act as allosteric integrase inhibitors by binding to the LEDGF/p75 interaction site on integrase (commonly referred to as the “LEDGF/p75 binding pocket” or “dimer interface”), thereby disrupting the protein–protein interaction between integrase and the integrase-binding domain (IBD) of LEDGF/p75, which mediates tethering of the pre-integration complex (PIC) to transcriptionally active chromatin.

In the presence of LEDGINs, HIV-1 proviral integration is consequently redirected toward less transcriptionally active genomic regions, including intergenic and heterochromatic domains, as well as loci distal to actively transcribed genes and regions enriched in H3K36me2/3 [23,24,25] (Figure 6B). This redistribution results from disruption of LEDGF/p75-mediated chromatin targeting via its PWWP domain, which normally recognizes H3K36me2/3 marks [137].

Under these conditions, integrase may rely on residual low-affinity chromatin interactions, such as binding to condensed or AT-rich DNA regions. This shift away from gene-dense and highly transcribed regions favors the establishment of a more repressive epigenetic environment, thereby limiting proviral transcription and reducing reactivation potential. Consequently, a larger fraction of proviruses becomes locked in a state of deep latency, characterized by persistently low viral RNA expression and reduced inducibility [23,24,25].

**Figure 7:**
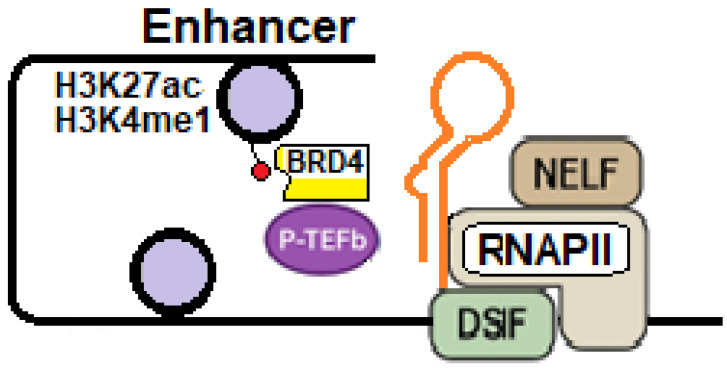
Role of BRD4 in Enhancer-driven Activation of HIV-1 Transcription. The HIV-1 provirus can integrate near active enhancer elements that influence proviral transcription. BRD4 is recruited to enhancer-associated regions through its bromodomains, which recognize acetylated histones such as H3K27ac and H3K4me1. At these sites, BRD4 facilitates transcriptional activation by recruiting and activating positive transcription elongation factor b (P-TEFb), thereby promoting the release of RNA polymerase II from promoter-proximal pausing and enabling productive transcriptional elongation.

Although LEDGINs redirect viral integration away from H3K36me2/3-marked chromatin, a small subset of cells with high levels of viral RNA expression can still persist. Vansant et al. showed that these high-viral-RNA-expressing cells were integrated in proximity to enhancer elements, regulatory genomic regions characterized by histone marks such as H3K27 acetylation (H3K27ac) and H3K4 monomethylation (H3K4me1). Integration near enhancers occurs largely stochastically and is independent of LEDGF/p75. As a consequence, the frequency of enhancer-proximal integration is not significantly altered by LEDGIN treatment. Enhancer-driven transcription is critically dependent on BRD4, which recognizes acetylated histones and facilitates the recruitment of transcriptional elongation machinery. When HIV-1 integrates near enhancer regions, BRD4 can sustain viral transcription despite LEDGF/p75 inhibition (Figure 7). This mechanism provides a plausible explanation for the residual HIV-1 transcription observed following LEDGIN treatment and represents an alternative pathway for viral gene transcription.

Recent evidence indicates that simultaneous modulation of these complementary pathways results in additive transcriptional silencing [18]. Specifically, the combination of LEDGINs, which redirect integration away from active gene bodies, with BRD4 modulators, which suppress enhancer-driven transcription, more effectively restricts HIV-1 gene expression than either strategy alone. This dual approach limits both LEDGF/p75-dependent and enhancer-associated transcriptional activation routes, thereby reinforcing transcriptional repression of the provirus.

Molecules belonging to the class of LEDGINs include CX14442, BI-1001, GS-9822, BI-224436, STP0404, BDM-2, DW-D-5, Ebselen, CX04328, CHIBA-3053, CHI-104, and the 3-hydroxypicolinamide class [24,138,139,140,141,142,143,144,145,146,147,148,149,150,151,152]. Some of these compounds, such as pirmitegravir and BDM-2, have been evaluated in preclinical studies and have advanced to clinical trial phases.

The research compound CX14442, a potent LEDGIN targeting the LEDGF/p75–integrase interaction, has been shown in vitro to retarget residual HIV-1 integration away from active genes into repressive chromatin and to increase both immediate latency and resistance to reactivation of residual proviruses, consistent with a block-and-lock–like phenotype in cell culture models. Although it has not been developed clinically and has not yet proven functional cure in vivo, CX14442 provides preclinical evidence that disrupting the LEDGF/p75–integrase interaction can contribute to a latent reservoir that is more refractory to reactivation [142].

BI-224436 acts through a dual allosteric mechanism of action: (i) inhibition of integrase-viral DNA assembly; (ii) blockade of IN-LEDGF/p75 interaction. However, clinical studies with BI-224436 were discontinued despite promising results. BDM-2 and pirmitegravir share critical structural analogies with BI-224436, but with novel chemical scaffolds with enhanced potency [143].

Although BDM-2 and pirmitegravir have primarily been developed as antiviral agents, both compounds belong to the class of LEDGINs targeting the integrase–LEDGF/p75 interaction. Given the established role of LEDGF/p75 in directing HIV-1 integration toward transcriptionally active chromatin, it can be hypothesized that these compounds may also influence proviral integration site selection and contribute to a block-and-lock–like phenotype. However, direct experimental evidence supporting such an effect is currently lacking.

Pirmitegravir is a pyrrolopyridine-based allosteric HIV-1 integrase inhibitor (ALLINI) that has demonstrated picomolar potency in vitro with a >24,000 therapeutic index and has advanced to phase 2a clinical trials [138,144]. X-ray crystallographic and biochemical analyses have revealed that pirmitegravir binds to the LEDGF/p75 binding pocket on the HIV-1 integrase (IN) catalytic core domain (CCD) dimer. The compound acts as a molecular glue, engaging a triad of invariant CTD residues (Tyr226, Trp235, Lys266) to nucleate an ectopic CCD-CTD interaction [145]. This induces aberrant IN hypermultimerization, which blocks the IN-viral RNA interaction and disrupts proper localization of HIV-1 RNA genomes in viral particles during maturation [138,145]. Importantly, pirmitegravir potently inhibits both wild-type HIV-1 and all drug-resistant viral phenotypes that have emerged against currently used therapies (INSTIs, NRTIs, NNRTIs, PIs), making it a promising option for heavily treatment-experienced patients [144]. Extensive preclinical pharmacological and toxicity investigations have been conducted [138]. Pharmacokinetic studies demonstrated favorable drug-like properties, making the compound suitable for clinical development. Toxicity assessments showed outstanding safety properties across multiple animal models. Pirmitegravir has progressed through clinical Phase 1 and Phase 2a. Phase 1: a single-ascending-dose study was completed in healthy volunteers, establishing initial human pharmacokinetics and safety [138]. Phase 2a: currently advancing in clinical trials evaluating antiviral efficacy in HIV-infected patients [144]. As of the most recent reports, pirmitegravir represents the most clinically advanced ALLINI in development, being described as a “first-in-class ALLINI that targets LEDGF/p75 binding site and has advanced to a human trial” [138,146].

BDM-2 has completed a single-ascending-dose phase I clinical trial (NCT03634085), with results showing that the compound is well-tolerated in healthy volunteers. No antagonism was observed between BDM-2 and a panel of 16 clinical antiretrovirals, and the compound retained high antiviral activity against HIV-1 variants resistant to integrase strand transfer inhibitors and other antiretroviral classes. The virologic profile and safety data from the phase I trial support further clinical investigation of BDM-2 in combination regimens. No serious adverse events or dose-limiting toxicities have been reported in the available clinical trial data [147].

Although preliminary efficacy of STP0404 and BDM-2 in humans is supported by their potent antiviral activity in vitro and favorable safety profiles, further studies are needed to determine whether these two agents, in combination with standard cART, can delay HIV-1 viral load rebound after treatment interruption.

GS-9822 is a potent LEDGIN that retargets HIV-1 proviral integration toward less transcriptionally active chromatin regions and promotes viral latency. GS-9822 has demonstrated superior efficacy compared with CX14442, but STP0404 exhibits superior potency and retargeting activity at picomolar concentrations, with a therapeutic index exceeding 24,000 [23].

Detailed human pharmacokinetic parameters—including absorption, distribution, metabolism, and excretion—as well as bioavailability data, are not available in the current medical literature because GS-9822 remains a preclinical candidate and has not progressed to human clinical trials [23]. The safety profile of GS-9822 is characterized by species-specific urothelial toxicity observed in cynomolgus monkeys, but not in rats [152].

## 7. Emerging Epigenetic RNA-Based and Gene Therapy–Mediated Block-and-Lock Strategies

### 7.1. Noncoding RNAs as Block-and-Lock Approach

Long noncoding RNAs (lncRNAs) are emerging regulators of HIV-1 latency through epigenetic and post-transcriptional mechanisms. Among them, Metastasis-Associated Lung Adenocarcinoma Transcript 1 (MALAT1) has been identified as a promoter of HIV-1 reactivation rather than silencing. Mechanistically, MALAT1 displaces the Polycomb Repressive Complex 2 (PRC2) from the HIV-1 LTR promoter, reducing H3K27me3 deposition and promoting viral transcription [167]. Consistently, MALAT1 expression correlates positively with viral replication, whereas its knockdown suppresses HIV-1 transcription and replication in multiple experimental models [168,169].

Different silencing strategies, including antisense oligonucleotides, siRNA, and CRISPR-Cas9, have consistently shown that MALAT1 downregulation reduces viral transcription, enhances antiviral innate immune responses, and limits HIV replication [168]. Although these approaches have not been explicitly developed within a block-and-lock framework, their mechanisms are fully consistent with this strategy, as MALAT1 downregulation is expected to preserve PRC2-mediated epigenetic repression at the viral promoter and stabilize deep latency. However, clinical translation remains limited, and efficient delivery systems will be required to achieve durable silencing in vivo.

In contrast, Nuclear Paraspeckle Assembly Transcript 1 (NEAT1) inhibits HIV-1 replication by retaining viral transcripts within nuclear paraspeckles and limiting mRNA export. Its overexpression could, in principle, complement block-and-lock strategies, although its physiological downregulation during T-cell activation may be a limiting factor [170].

### 7.2. HIV Antisense RNA (AST/ASP RNA) for Block-and-Lock Approaches

Among lncRNA-based approaches, HIV-1 antisense RNA (AST/ASP RNA) represents a particularly promising mechanism for durable viral silencing. Early studies demonstrated that ASP RNA promotes proviral latency by recruiting PRC2 to the HIV-1 LTR, increasing H3K27me3 deposition, reducing RNA polymerase II binding, and suppressing viral transcription [171]. Subsequent work showed that antisense transcription is associated with resistance to latency reversal, indicating a dominant role in maintaining a non-reactivatable state [153].

More recently, ectopic expression of AST in primary CD4+ T cells from individuals on cART was shown to suppress viral reactivation induced by both pharmacological and T cell receptor stimuli, providing direct evidence for its potential as a block-and-lock agent [154]. Mechanistically, AST promotes viral silencing through PRC2 recruitment, chromatin compaction, transcriptional interference, and interaction with host inhibitory factors.

Notably, unlike host-derived lncRNAs, HIV antisense RNA specifically targets the viral genome, minimizing off-target effects on host gene expression. Its ability to induce heritable epigenetic silencing and confer resistance to latency reversal further supports its potential as a durable block-and-lock strategy [155,171].

From a translational perspective, stable expression of antisense RNA could be achieved through lentiviral vector–based gene therapy approaches. Advances in pseudotyping strategies enable targeted delivery to CD4+ T cells, including resting memory subsets that constitute the major HIV reservoir, supporting the feasibility of long-term silencing following limited administrations [156,157].

Overall, RNA-based and gene therapy–mediated approaches represent a mechanistically distinct class of block-and-lock strategies. While still in a preclinical stage, their ability to induce targeted, potentially durable epigenetic silencing highlights their relevance to the development of curative HIV-1 interventions.

## 8. Redirecting HIV-1 Integration into LADs: CPSF6 Knockdown and Capsid Inhibitors as Block-and-Lock Strategies

Multiple lines of evidence indicate that Cleavage and Polyadenylation Specificity Factor subunit 6 (CPSF6) is a critical host factor for HIV-1 integration, guiding the PIC to nuclear speckles and to gene-dense, transcriptionally active regions. When CPSF6 is depleted or its interaction with the HIV-1 capsid is disrupted, integration is redirected away from these active regions and toward heterochromatic LADs at the nuclear periphery, which are known to be transcriptionally repressive. Specifically, Li et al. mapped millions of integration sites and found that CPSF6 knockout in human cells resulted in a marked increase in integration events within LADs, with a corresponding decrease in integration within speckle-associated domains (SPADs), which are euchromatic and transcriptionally active [172]. Engelman further corroborated that the absence of CPSF6-capsid interaction leads to mis-targeting of integration to LADs [31]. Other studies, in addition to those by Li et al. and Engelman et al., demonstrated that CPSF6 knockout or knockdown redirects proviral integration toward less transcriptionally active genomic regions and LADs. Achuthan et al. showed that loss of CPSF6-capsid interaction results in the HIV-1 PIC accumulating at the nuclear periphery and integrating into transcriptionally repressed lamina-associated heterochromatin, rather than gene-dense regions in the nuclear interior [27]. Sowd et al. further demonstrated that CPSF6 knockout or knockdown decreases integration into transcriptionally active genes and regions enriched for activating histone marks, with a corresponding shift toward less active chromatin [26]. Chaudhuri et al. confirmed that disruption of CPSF6-capsid binding redirects integration away from gene-dense, transcriptionally active regions into regions of low transcriptional activity [173].

### 8.1. Role of CPSF6 in Host Gene Regulation and HIV-1 Replication

CPSF6 is a host cellular protein involved in mRNA maturation, a fundamental process required for proper gene expression. In particular, CPSF6 contributes to the formation of the poly(A) tail, a structure that influences mRNA stability and functionality [174,175].

During HIV-1 infection, the virus exploits CPSF6 by directly binding to it via the viral capsid. This interaction is critical for viral replication, as it enables proper intracellular trafficking of the viral PIC and its transport into the nucleus. Through CPSF6, the viral genome is preferentially integrated into transcriptionally active, gene-rich regions of host chromatin, thereby promoting efficient viral replication [176,177,178].

Beyond its role in viral trafficking and genome integration, CPSF6 also modulates host gene expression. Upon recruitment by the viral capsid, CPSF6 is redistributed to nuclear speckles, specialized compartments enriched in factors involved in mRNA processing. In this context, CPSF6 regulates the selection of polyadenylation sites, altering the processing of host mRNAs [179]. This regulation determines the length of the 3′ untranslated regions (3′ UTRs) of cellular mRNAs, which contain key regulatory elements. By modulating 3′ UTR length, CPSF6 influences mRNA stability, localization, and post-transcriptional regulation, thereby reshaping host gene expression programs.

HIV-1 infection perturbs these processes. Through its interaction with CPSF6, the virus recapitulates aspects of alternative polyadenylation, which governs approximately 70% of host gene expression, resulting in a phenotype similar to that observed in CPSF6 knockout cells. This phenotype is characterized by altered mRNA stability, post-transcriptional regulation, and host gene expression, creating a cellular environment more permissive to HIV-1 replication [179].

In addition, the C-terminal short amino acid nuclear localization signal (NLS) in CPSF6 facilitates steps of HIV-1 infection following nuclear import by influencing the localization of the viral genome within the nucleus [180]. As previously reported, in the absence of CPSF6, the PIC fails to traffic to nuclear speckles and instead accumulates at the nuclear periphery, where integration events become enriched within LADs, which are transcriptionally repressive.

### 8.2. LADs Structure and Functions

**Figure 8:**
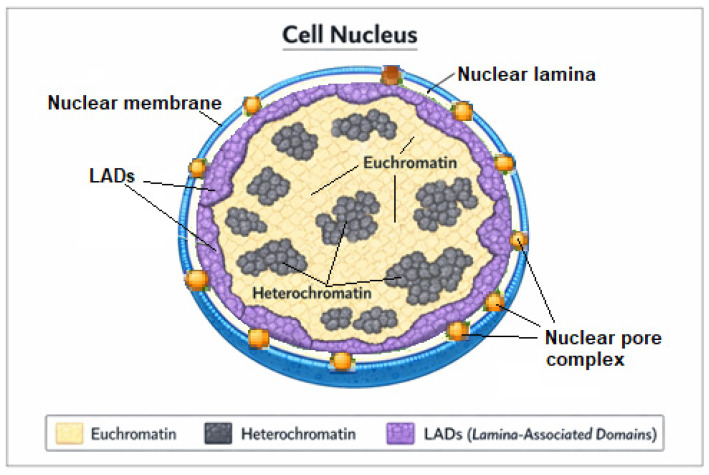
LADs, intranuclear heterochromatin, and euchromatin organization. LADs are illustrated as chromatin regions positioned in close proximity to and physically interacting with the nuclear lamina. LADs correspond to highly compacted heterochromatin, typically enriched in repressive histone modifications and characterized by absent or markedly reduced transcriptional activity. In addition to LADs, the figure also depicts heterochromatin domains that are not directly bound to the nuclear lamina and are located more internally within the nucleoplasm. In contrast, euchromatin is less condensed chromatin that extends toward the nuclear interior and corresponds to transcriptionally permissive genomic regions.

LADs are large genomic regions, typically spanning megabases, that are in close contact with the nuclear lamina, a fibrous network lining the inner face of the nuclear envelope. LADs are composed predominantly of heterochromatic chromatin, enriched in repressive histone modifications, primarily H3K9me2 and H3K9me3, with H3K27me3 marking specific LAD subtypes. These features contribute to low chromatin accessibility and transcriptional repression (Figure 8, Table 3) [181,182].

The primary functions of LADs include regulating nuclear architecture, repressing gene expression, and controlling chromatin accessibility. Lamina association contributes to spatial genome compartmentalization, restricting gene expression within LADs and supporting overall chromosome organization during interphase. LADs largely correspond to the B compartment of the genome, characterized by compact chromatin and low transcriptional activity. However, not all B compartment regions are LADs, indicating that lamina association provides an additional layer of three-dimensional genome regulation. High-resolution mapping and integrative analyses have shown that while most LADs reside within the B compartment, some B compartment regions do not associate with the lamina, including polycomb-enriched domains marked by H3K27me3, which can occupy internal nuclear positions and remain transcriptionally repressed independently of stable lamina tethering [182,183,184]. LADs are dynamic structures whose position and composition change during the cell cycle, during differentiation, or in response to environmental cues. At the same time, lamina association can act as a mechanism of long-term epigenetic silencing, contributing to stable gene expression programs and cellular epigenetic memory [29,185]. LAD boundaries (the transition zone where lamina-associated, repressive chromatin switches to more internal, transcriptionally active chromatin) are often enriched in architectural and regulatory factors, such as CTCF and, in some contexts, YY1, which help define domain limits and maintain the separation between transcriptionally active regions and repressive peripheral chromatin domains [186].

### 8.3. LADs and HIV-1 Integration

Although LADs are generally less accessible due to condensed chromatin, HIV-1 integrase retains the ability to catalyze strand transfer into these regions. This process is likely facilitated by local DNA flexibility and nucleosome dynamics, which allow HIV-1 integrase to access and integrate viral DNA even within transcriptionally repressive chromatin environments, including LADs [187]. The absence of CPSF6 does not alter integrase enzymatic activity but alters the spatial distribution of the PIC, increasing the relative enrichment of integration events within LAD-enriched, lamina-proximal genomic regions [27,141,180]. Recently, we proposed a possible explanation for why HIV-1 capsids unbound to CPSF6 could contribute to increased interactions between CPSF6-unbound HIV-1 capsids and lamina-proximal chromatin, thereby favoring integration within LAD-enriched regions [4], based on the work of Emerson et al. on the Gmr1-like family of Gypsy/Ty3-like retrotransposons in the ancestor of amniotes [188]. We suggested that the SCAN domain, found in many C2H2-type zinc finger proteins and derived from the C-terminal portion of the gag capsid protein, may mediate protein–protein interactions with retroviral capsids [4]. These zinc finger proteins appear to be more prevalent in LADs, potentially facilitating proviral integration into these regions when the capsid is not bound to CPSF6, consistent with the evolutionary relationship described by Emerson et al. The idea that SCAN domain-containing zinc finger proteins, which are enriched in LADs, could interact with unbound HIV-1 capsids and facilitate integration into these regions remains speculative and warrants further research into host–virus interactions [4].

HIV-1 proviruses integrated into LADs are less likely to be transcribed due to the repressive chromatin environment, which impedes the access of transcriptional machinery and limits RNAPII activity (Table 3). This chromatin context is associated with a higher propensity to establish and maintain HIV-1 latency, as these proviruses are less responsive to activation signals and latency-reversing agents.

Zheng et al. demonstrated that CPSF6 deficiency not only redirects HIV-1 integration but also impairs transcriptional reactivation by dysregulating CDK9 and RNAPII phosphorylation in primary CD4+ T cells [189]. Specifically, CPSF6 knockout leads to abnormal stabilization of the protein phosphatase 2A (PP2A). This stabilization increases PP2A activity, which in turn dephosphorylates CDK9. Reduced CDK9 phosphorylation impairs its ability to phosphorylate the CTD of RNA polymerase II, a critical step for transcriptional elongation and HIV-1 latency reversal. As a result, latent HIV-1 proviruses in CPSF6-deficient cells show markedly reduced transcriptional reactivation in response to latency-reversing agents such as PMA. The study further demonstrates that pharmacological inhibition of PP2A with LB100 restores CDK9 and RNA polymerase II phosphorylation and rescues HIV-1 transcription in CPSF6 knockout cells, confirming the mechanistic link. This regulatory pathway is independent of CPSF6′s role in integration site selection and mRNA cleavage/polyadenylation.

In light of these observations, a critical question arises: is a block-and-lock therapeutic strategy based on pharmacological inhibition of CPSF6 safe? Disruption of CPSF6 is known to exert pleiotropic effects on host cell gene regulation and immune function, including widespread alterations in alternative polyadenylation [176,177,179]. Collectively, these findings raise significant concerns about the feasibility and safety of directly targeting CPSF6 within a block-and-lock therapeutic approach.

An alternative strategy may involve preventing the interaction between CPSF6 and the viral capsid rather than depleting CPSF6 itself. Such an approach could impair PIC nuclear transport and favor integration into LADs, while preserving the cellular CPSF6 pool and its physiological functions. In this context, Bester et al. showed that the HIV-1 capsid inhibitor GS-6207 (lenacapavir) binds to the interface between two adjacent capsid subunits and enhances both intra- and inter-hexamer interactions, thereby stabilizing the curved capsid lattice. This stabilization interferes with the binding of host cofactors such as Nup153 and CPSF6, which are required for efficient nuclear import and for directing viral integration toward gene-rich chromatin regions [28].

**Figure 9:**
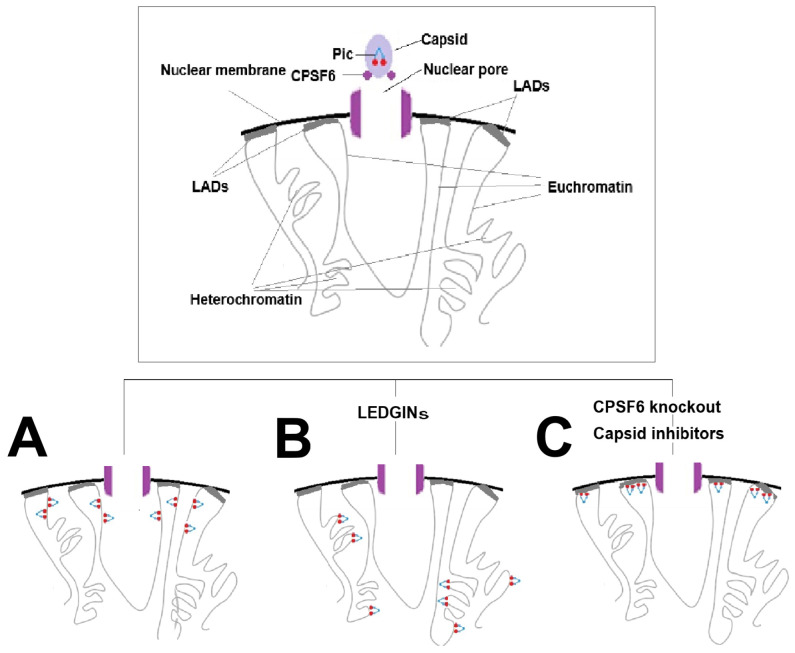
Schematic representation of HIV-1 proviral integration in the absence of drugs (**A**), in the presence of LEDGINs (**B**), and following CPSF6 knockout or treatment with lenacapavir (**C**). Under basal conditions (**A**), HIV-1 integrates preferentially within gene-dense, transcriptionally active euchromatin, although a measurable fraction of events occurs in transcriptionally repressed heterochromatin. LEDGIN treatment (**B**) redirects integration away from active euchromatin toward gene-poor, transcriptionally repressed heterochromatin located within the nuclear interior. In contrast, CPSF6 knockout or disruption of the capsid–CPSF6 interaction by lenacapavir (**C**) alters nuclear trafficking and promotes integration at the nuclear periphery, particularly within LADs.

As a result, GS-6207 substantially reduced integration in gene-dense regions and conversely, enhanced integration in LADs. These observations mirror the outcomes seen after CPSF6 knockout or knockdown, in which disruption of CPSF6–capsid interactions likewise shifts HIV-1 integration away from transcriptionally active euchromatin and toward LADs (Figure 9). However, the changes induced by the inhibitor were less pronounced than those observed following CPSF6 knockout, indicating that GS-6207 may not completely displace this host cofactor [28]. Other scientific studies support the findings of Bester et al., demonstrating that lenacapavir binds to a conserved hydrophobic pocket in the HIV-1 capsid hexamer that overlaps with the binding site used by host factors such as CPSF6 and Nup153, thereby competing with—but not necessarily fully abolishing—their interaction with the capsid that normally directs the PIC to transcriptionally active, gene-rich regions [31,190,191,192].

Importantly, lenacapavir has already been approved for clinical use in the treatment of HIV-1 infection. Under the brand name Sunlenca, the U.S. Food and Drug Administration (FDA) granted approval for lenacapavir in combination with other antiretrovirals as a twice-yearly treatment option for heavily treatment-experienced adults with multidrug-resistant HIV-1 infection, and marketing authorizations have been granted in multiple regions. In addition, lenacapavir has received regulatory endorsements for HIV-1 prevention as a long-acting injectable administered biannually. These approvals reflect a substantial body of clinical evidence supporting the safety and efficacy of lenacapavir in the management of HIV-1 infection, underscoring its potential as a clinically viable component of novel therapeutic strategies that leverage capsid inhibition to suppress viral replication [30,32,33,34,35].

### 8.4. Could Capsid–CPSF6 Interaction Inhibitors Enable a More Stable Block-and-Lock Strategy than LEDGINs?

HIV-1 integration site selection is increasingly recognized as a critical determinant of long-term proviral transcriptional fate and, consequently, as a potential leverage point for block-and-lock strategies. Although multiple host cofactors contribute to integration targeting, their relative importance in establishing durable proviral silencing remains incompletely defined. In this context, the distinct and non-redundant roles of CPSF6 and LEDGF/p75 raise important considerations for therapeutic intervention.

Accumulating evidence, including the work of Sowd et al. [26], indicates that CPSF6 plays a central role in directing HIV-1 integration toward gene-dense, transcriptionally active chromatin. In contrast, LEDGF/p75 appears to predominantly influence the positioning of integration within gene bodies, rather than governing higher-order chromatin compartment selection. Pharmacological disruption of the LEDGF/p75–integrase interaction through LEDGINs shifts integration away from active euchromatin toward gene-poor, transcriptionally repressed regions (Table 3). Notably, LEDGIN treatment has been associated with proviruses localizing deeper within the nuclear interior, in heterochromatin-enriched environments (Figure 9).

By comparison, CPSF6 knockout—or disruption of the capsid–CPSF6 interaction, for example by lenacapavir—impairs proper nuclear trafficking of the PIC. This alteration results in a redistribution of integration events toward the nuclear periphery, particularly within LADs (Figure 9, Table 3) [26,27]. Thus, although both LEDGIN treatment and CPSF6 depletion reduce integration within transcriptionally active chromatin, the resulting chromatin landscapes are not equivalent (Figure 9).

Importantly, LADs are generally regarded as more stable and deeply repressive than the internal heterochromatin favored under LEDGIN treatment. The heterochromatic regions targeted in the presence of LEDGINs are enriched in repressive histone modifications, such as H3K9me3, yet are not necessarily anchored to the nuclear lamina. While these regions are less transcriptionally permissive and are associated with reduced basal proviral expression, they often retain a degree of chromatin plasticity and may remain susceptible to reactivation.

In contrast, loss of CPSF6 function redirects integration toward chromatin domains that are stably associated with the nuclear lamina (Figure 9). These LADs are characterized by high levels of H3K9me2/3, low gene density, enrichment within the nuclear B compartment, and a compact, structurally constrained chromatin environment (Table 3). Collectively, these features define some of the most transcriptionally repressive regions of the nuclear genome. From a transcriptional perspective, integration within LADs following CPSF6 knockdown or knockout is therefore generally considered more repressive than integration within the internal heterochromatin promoted by LEDGINs.

**Figure 10:**
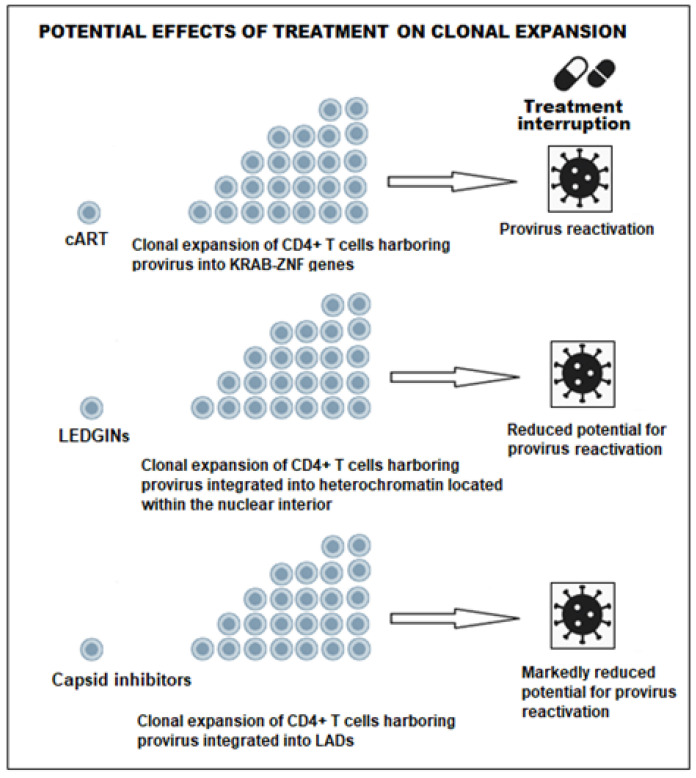
Potential effects on the clonal expansion of infected CD4+ T cells in patients receiving prolonged cART (including integrase inhibitors, nucleoside and non-nucleoside reverse transcriptase inhibitors, and protease inhibitors), LEDGINs, and capsid inhibitors.

From a block-and-lock standpoint, these observations raise the possibility that targeting CPSF6-dependent integration pathways with capsid inhibitors could more effectively bias proviral integration toward highly silenced chromatin compartments, such as LADs, than strategies that act exclusively on LEDGF/p75 (Figure 10). However, it is important to emphasize that this hypothesis remains largely theoretical. Direct experimental evidence demonstrating that capsid inhibition leads to durable proviral silencing through altered integration targeting is currently lacking. Nevertheless, the hierarchical role of CPSF6 in regulating integration site selection provides a compelling rationale to further investigate the capsid–CPSF6 axis as a potential, albeit indirect, strategy for block-and-lock interventions. Future studies will be required to determine whether modulation of this pathway can meaningfully contribute to the establishment of a deeply latent, stable, and non-reactivable HIV-1 reservoir.

HIV-1 preferentially integrates into actively transcribed genes, but in patients on long-term cART, intact, latent proviruses are commonly found within Krüppel-associated box zinc finger (KRAB-ZNF) genes. These regions are characterized by heterochromatin, leading to silenced, non-productive infection and the survival of infected T-cell clones, contributing significantly to the viral reservoir [193,194,195]. Conversely, cells with proviruses in transcriptionally active regions are prone to viral expression and elimination. This natural selection results in an enrichment of cells harboring proviruses in repressive regions after years of cART. Importantly, despite being silenced, proviruses integrated into KRAB-ZNF genes can undergo clonal expansion and produce virus upon appropriate stimulation, demonstrating that reactivation remains possible [193,194,195].

LADs share key features with heterochromatin and KRAB-ZNF genes, including enrichment in repressive histone marks (H3K9 di- and trimethylation) and in repetitive DNA elements that serve as platforms for zinc finger proteins, all of which contribute to strong transcriptional repression [195]. There is currently no direct and definitive experimental evidence that HIV-1 proviruses integrated into LADs are no longer reactivatable. However, some observations suggest that proviruses integrated into LADs could indeed be stably silenced. Battivelli et al. observed that latent proviruses that are not readily reactivatable in a shock-and-kill strategy are more extensively integrated into LADs [196]. In addition, elite controllers, a rare subset of people living with HIV-1 who can naturally control the virus without cART, show significant enrichment of intact proviruses in LADs, particularly when clonally expanded proviruses are counted as independent integration events, suggesting that integration into these regions may strongly suppress viral transcription [197].

From a conceptual standpoint, distinct chromatin environments contribute different layers of transcriptional repression that influence the stability of integrated viral genomes. KRAB–ZNF gene clusters and LADs represent two such repressive genomic contexts, yet they operate through partially distinct mechanisms.

KRAB–ZNF clusters primarily repress transcription through a molecular silencing pathway mediated by the KRAB/KAP1 (TRIM28) complex. The KRAB domain recruits KAP1, which in turn engages SETDB1 and other chromatin-modifying enzymes, leading to the deposition of H3K9me3 and the establishment of HP1-enriched heterochromatin. This mechanism generates a robust epigenetic barrier to transcription and is particularly effective in silencing transposable elements and exogenous DNA sequences. Thus, integration within KRAB–ZNF regions predominantly subjects the proviral genome to strong molecular repression driven by heterochromatin formation [198].

In contrast, LADs provide a dual-layered repression. Similar to KRAB–ZNF clusters, LADs are enriched in repressive histone marks such as H3K9me2/3 and are generally transcriptionally inactive. However, LADs additionally confer spatial sequestration through physical association with the nuclear lamina. This peripheral positioning limits access to transcriptional machinery, reduces enhancer–promoter contacts, and reinforces chromatin compaction within a structurally constrained nuclear compartment. Consequently, LAD-associated integration couples epigenetic silencing with three-dimensional genome organization, potentially creating a more stringent barrier to reactivation [29,199].

Finally, viral genomes different from HIV-1 can associate with repressive nuclear compartments and adopt a transcriptionally silent state. During latency, the Epstein–Barr virus (EBV) genome persists as a chromatinized episome within the nucleus of infected cells and can localize to regions near the nuclear periphery. Evidence indicates that EBV episomes may interact with components of the nuclear lamina, including lamin B1 and lamin A/C, and that this spatial positioning correlates with the enrichment of repressive histone modifications such as H3K9me2 and H3K9me3, which contribute to silencing of the majority of viral genes during latency [200,201,202]. During latency, only a restricted subset of viral genes required for persistence remains expressed, whereas most of the viral genome is epigenetically silenced [202,203,204]. Perturbation of this repressive chromatin landscape can facilitate viral reactivation and entry into the lytic cycle.

Other herpesviruses, such as Kaposi’s sarcoma–associated herpesvirus (KSHV), also persist as chromatinized episomes that acquire repressive histone marks during latency [205,206,207]. In KSHV, the latency-associated nuclear antigen (LANA) tethers the viral episome to host chromosomes and contributes to the maintenance of latent infection. Although KSHV genomes can associate with repressive chromatin environments, stable or obligate localization within LADs has not been definitively established.

Based on these observations, we proposed a theoretical model in which prolonged treatment with capsid inhibitors that favor HIV-1 integration into LADs gradually selects for CD4+ T cells harboring proviruses in these repressive domains, where transcriptional silencing is maintained, and the risk of viral reactivation is minimized (Figure 10) [4]. In this context, capsid inhibitors would not only block new rounds of viral replication but also, through integration site–driven selection, indirectly promote long-term proviral silencing, potentially reducing viral rebound following therapy interruption. It is important to emphasize that this model remains entirely hypothetical. Its validation would require long-term clinical studies with extended treatment with capsid inhibitors. Nevertheless, this framework provides a valuable conceptual advance for block-and-lock strategies, highlighting that both the epigenetic environment and the genomic context of proviral integration, combined with clonal selection over time, may be critical for achieving durable HIV-1 latency.

### 8.5. CFIm, Alternative Polyadenylation Remodeling by HIV-1, and Implications for Capsid Inhibitors in Block-and-Lock Strategies

The mammalian cleavage factor I (CFIm) complex is a key regulator of alternative polyadenylation, a process that determines the length of the 3′ untranslated region (3′ UTR) in most human mRNAs. CFIm promotes the use of distal polyadenylation sites, thereby generating transcripts with longer 3′ UTRs [208]. Although the 3′ UTR does not encode proteins, it contains regulatory elements that control mRNA stability, translation, export, and localization. Therefore, longer 3′ UTRs enable more precise post-transcriptional regulation of gene expression.

When CFIm components such as CPSF5 or CPSF6 are depleted, polyadenylation shifts toward proximal sites, leading to widespread 3′ UTR shortening and reduced regulatory control, with significant effects on cellular gene expression [208].

HIV-1 interferes with this pathway through a capsid-dependent mechanism. During early infection, the viral capsid binds CPSF6 and relocalizes it to nuclear speckles, disrupting CFIm function and inducing global alternative polyadenylation remodeling. In infected CD4+ T-cells and permissive cell lines, wild-type HIV-1 promotes proximal polyadenylation and the accumulation of transcripts with shortened 3′ UTRs. In contrast, capsid mutants unable to bind CPSF6 fail to induce these changes, demonstrating a direct link between capsid–CPSF6 interaction and host transcriptome remodeling [176,208,209].

Capsid inhibitors such as lenacapavir disrupt the interaction between the viral capsid and CPSF6 and, at least in principle, could preserve CFIm localization and function, potentially preventing HIV-1–induced dysregulation of alternative polyadenylation. However, this remains a theoretical model. To date, no direct experimental studies have demonstrated that lenacapavir effectively prevents the alternative polyadenylation remodeling induced by HIV-1 infection. Although biologically plausible and consistent with current knowledge of capsid–CPSF6 interactions, this effect has not yet been experimentally validated. If confirmed, this mechanism could have important implications for block-and-lock strategies. Beyond inhibiting viral replication, capsid inhibitors might help maintain a more transcriptionally stable and less permissive cellular environment by limiting HIV-1–driven transcriptomic reprogramming and cellular dysregulation, processes that may contribute to viral persistence and reactivation. Thus, while requiring experimental confirmation, this additional layer of action could provide a unique advantage for capsid inhibitors over other antiretroviral drug classes, potentially supporting strategies aimed at a long-term functional cure.

### 8.6. Capsid-Centered Modulation of HIV-1 Integration: Current Limits and Future Directions Toward LAD Targeting

Although lenacapavir provides the first proof-of-concept that pharmacological manipulation of the capsid can alter integration site selection, its effects overlap only partially with those of CPSF6 depletion. Recent structural studies show that lenacapavir can bind to unoccupied hydrophobic pockets in capsid hexamers without fully displacing CPSF6, which binds through its low-complexity regions to create multivalent interactions with the capsid lattice [28,210]. This likely permits residual CPSF6-mediated influences on trafficking toward gene-rich chromatin. Moreover, lenacapavir stabilizes the capsid lattice allosterically rather than directly removing CPSF6. In contrast, CPSF6 knockout eliminates all CPSF6-dependent guidance, resulting in a more pronounced redistribution of viral complexes away from SPADs and an increased probability of integration within LAD-enriched regions [26,27,28].

Based on the currently available literature, no experimental compound has yet been reported to redirect HIV-1 integration more effectively toward LAD-enriched regions than lenacapavir. Nevertheless, several promising research avenues are emerging [211,212]. New capsid-targeting agents (e.g., KFA-027 and additional GS-6207 analogs) are being developed primarily to overcome resistance, but their altered binding modalities or enhanced perturbation of capsid conformation could, in theory, more strongly disrupt CPSF6–capsid interactions. Although no published data currently confirm this, the structural diversity of these molecules may provide unforeseen opportunities to strengthen LAD-biased integration. In addition, small molecules or gene-modifying approaches that modulate CPSF6 function might more closely approximate selected aspects of the CPSF6 knockout phenotype. Despite their conceptual appeal, such strategies face significant toxicity and specificity challenges and therefore remain primarily theoretical.

Perhaps the most realistic near-term approach involves combining capsid inhibitors with agents that reinforce transcriptional repression, such as BRD4 modulators or chromatin-targeting compounds.

The clinical availability of lenacapavir provides an important opportunity to investigate its effects on HIV-1 integration. Its demonstrated ability to bias viral integration away from gene-dense chromatin and toward LAD regions suggests that prolonged administration could gradually enrich for a transcriptionally repressed reservoir, potentially steering the virus toward a functionally silent state.

Although entirely speculative at this stage, this model merits rigorous investigation. If validated, capsid-based manipulation of integration-site selection could become an important component of future block-and-lock cure strategies.

## 9. Conclusions

Despite significant advances in understanding HIV-1 latency, clinically effective block-and-lock strategies remain elusive. Approaches based on Tat inhibitors, BRD4 modulators, CRISPR–Cas technologies, splicing inhibitors, and cyclin-dependent kinase inhibitors have demonstrated efficacy in experimental systems but have not yet reached clinical application due to limited potency, off-target effects, toxicity, or formulations unsuitable for long-term administration. In addition, many of these approaches rely on modulation of host factors, raising important safety concerns, particularly for strategies that would require sustained or irreversible effects.

Integration site modulators may represent a potentially important class of compounds for block-and-lock strategies. This class currently includes LEDGINs, and experimental evidence suggests that capsid inhibitors such as lenacapavir—shown to bias viral integration toward LADs—may be considered part of this category. Some of these compounds have entered Phase I and II clinical trials, and lenacapavir is already in clinical use.

Prolonged antiretroviral therapy combined with integration site modulation could gradually enrich the reservoir for proviruses integrated into transcriptionally silent chromatin, whereas proviruses in more permissive regions may be preferentially lost due to cytopathic effects or immune clearance. This could be particularly relevant for capsid inhibitors, which have been shown to bias proviral integration toward highly transcriptionally silent regions such as LADs. In this context, the combined effect of targeted integration and long-term selective pressure could progressively favor the persistence of deeply silenced proviruses, while more transcriptionally active integrations are eliminated over time. However, the duration of therapy required, the doses necessary to achieve integration retargeting, and the detectability of reservoir changes in patients remain unknown. Moreover, whether such approaches can induce a self-sustaining, treatment-independent state—consistent with a true functional cure—has yet to be determined.

A key limitation of these strategies is the need for drug presence during specific phases of the viral life cycle, particularly at the time of infection and integration. Given the unpredictable and infrequent reactivation of the viral reservoir, achieving effective temporal coverage would likely require sustained or repeated drug administration.

The ability of lenacapavir to mimic the effects of CPSF6 knockout by limiting the interaction between the viral capsid and CPSF6, thereby redirecting integration toward LADs, together with its clinical availability, offers an interesting opportunity to investigate block-and-lock strategies in a translational setting. Moreover, the development of highly competitive capsid inhibitors that directly disrupt capsid–CPSF6 interactions, and thus more faithfully recapitulate the effects of CPSF6 knockout, may further enhance preferential integration into LADs and potentially strengthen the efficacy of block-and-lock approaches.

Importantly, strategies that transiently modulate host–virus interactions at defined stages of the viral life cycle (e.g., integration) may offer a more favorable safety profile than those that require long-term inhibition of host factor function, although this hypothesis requires experimental validation. Factors such as individual variability, the composition of the viral reservoir, and other potential confounding influences will need to be addressed in future studies.

Overall, these findings provide a conceptual framework for how integration site modulation might contribute to durable HIV-1 latency. Nevertheless, whether these strategies can reliably translate into long-term proviral silencing and protection from viral rebound remains uncertain. Furthermore, most currently available agents are likely to function as long-acting antiretroviral therapies rather than truly curative interventions, as their effects may not persist after drug clearance. Beyond biological efficacy, any intervention proposed as curative must also satisfy essential translational criteria, such as affordability and scalability; however, these aspects remain largely undefined for most current block-and-lock strategies. While lenacapavir provides an initial example of how access may be ensured within specific healthcare systems, broader considerations regarding cost, global accessibility, and large-scale implementation remain to be addressed. Careful translational studies integrating virological, genomic, and clinical data will be essential to determine the potential of capsid inhibitors and related approaches to enhance block-and-lock strategies in the context of functional HIV-1 cure research.

## Figures and Tables

**Table 2:** Time to viral rebound after treatment cessation in vivo or drug removal in vitro ^1^.

Intervention	Time to Viral Rebound	References
cART alone	2–4 weeks (median time 16 days) in humans. 3–10 days in humanized mice of HIV-1 infection.	[5,40]
dCA + cART	10–19 days in humanized mice of HIV-1 infection.	[16,40]
Novel Tat inhibitors (1,3,4-oxadiazole derivatives, benzoxazole compounds, cyclic peptides, aromatic heterocycles)	No in vivo studies. No in vitro studies have determined the time required for transcriptional suppression after drug removal.	[44,45,46,47,48,49,50,51,52,53]
LLDT + cART	LLDT-8 + cART does not delay viral rebound in SIV-infected macaques after cART discontinuation.	[56,57]
Topotecan	No in vivo studies. In in vitro studies, suppression of HIV-1 reactivation persisted for ~72 h after the drug was removed from the culture medium.	[58]
CRISPR–Cas (Targeted provirus editing)	Quantitative rebound kinetics are not yet well defined.	[59,60,61,62,63]
EBT-101 infusion (CRISPR excising HIV DNA) + cART in humans	In humans, ~2–4 weeks; one individual, ~16 weeks.	[64]
SF3B1 inhibitors	No in vivo studies. In in vitro studies, suppression of HIV-1 reactivation by sudemycin D6 persisted for ~72 h after the drug was removed from the culture medium.	[65]
RNAi technologies	Quantitative rebound kinetics are not yet well defined.	[66,67,68,69,70,71,72,73,74,75,76]
ZL0580	~4 weeks in a humanized mouse model of HIV-1 infection.	[98]
NF-κB Inhibitors	No in vivo studies. Precise kinetics have not been systematically reported in standard in vitro latency models.	[100]
PI3K–AKT–mTOR Pathway Modulators	No in vivo studies. No in vitro studies have determined the time required for transcriptional suppression after drug removal.	[103,104]
Danusertib	No in vivo studies. No in vitro studies have determined the time required for transcriptional suppression after drug removal.	[106]
PF-3758309	No in vivo studies. No in vitro studies have determined the time required for transcriptional suppression after drug removal.	[109]
Protein kinase C inhibitors	No in vivo studies. No in vitro studies have reported the time course of transcriptional suppression following drug removal.	[110]
CDK9 Inhibitors	No in vivo studies. In in vitro studies, the transcriptional suppression persists for at least 24 h after drug removal.	[111]
CDK8/19 Inhibitors	No in vivo studies. In in vitro studies, the transcriptional suppression persists for at least 7 days after drug removal.	[111]
CDK7 Inhibitors	No in vivo studies. No in vitro studies have determined the time required for transcriptional suppression after drug removal.	[111]
SR Kinase Inhibitors	No in vivo studies. No in vitro studies have determined the time required for transcriptional suppression after drug removal.	[112]
H3K27 demethylase inhibitors, histone acetyltransferase (HAT) inhibitors	No in vivo studies. In in vitro studies, transcriptional suppression lasts less than 72 h after removal of GSK-J4.	[121,122,123,124]
CBL0100	No in vivo studies. No in vitro studies have determined the time required for transcriptional suppression after drug removal.	[128]
Q308	No in vivo studies. No in vitro studies have determined the time required for transcriptional suppression after drug removal.	[129]
LEDGINs	No in vivo or in vitro studies calculating the time of transcriptional suppression after drug removal.	[138,139,140,141,142,143,144,145,146,147,148,149,150,151,152]
HIV Antisense RNA (AST/ASP RNA)	No in vivo studies. In in vitro studies, AST suppresses HIV-1 transcription and blocks reactivation in response to stimuli under the experimental conditions of AST expression. No quantitative data have been published measuring how long the suppression persists following AST expression in vitro.	[153,154,155,156,157]

^1^ It should be emphasized that measuring viral rebound after drug withdrawal in vitro is not equivalent to assessing time to viral rebound in vivo, which requires reactivation, production, and systemic spread of replication-competent virus in an animal model or in patients following treatment interruption. Nevertheless, given the limited number of in vivo studies evaluating candidate block-and-lock compounds, we have also reported in vitro findings obtained after removal of the tested compounds, either alone or in combination with antiretroviral therapy, for all compounds that have not yet been tested in vivo.

**Table 3:** Comparative epigenomic and structural features of major chromatin states. Arrow symbols indicate the relative levels of the indicated variables: one to three upward arrows (↑–↑↑↑) denote increasing levels or enrichment, whereas one to three downward arrows (↓–↓↓↓) denote decreasing levels or reduced expression.

Chromatin Type	H3K9me2/3	Gene Density	Enrichment in Nuclear B Compartment *	Compact/Structurally Constrained Environment
Euchromatin	↓	↑↑↑	**↓**	**↓**
Intranuclear heterochromatin	↑↑	↓↓	↑↑	↑↑
LADs	↑↑↑	↓↓↓	↑↑↑	↑↑↑

* The B compartment represents regions of the genome that are transcriptionally inactive, gene-poor, heterochromatic, and spatially segregated within the nucleus.

## Data Availability

No new data were created or analyzed in this study. Data sharing is not applicable to this article.
